# Identifying Barriers and Enablers for Nurse‐Initiated Care for Designing Implementation at Scale in Australian Emergency Departments: A Mixed Methods Study

**DOI:** 10.1111/jocn.17693

**Published:** 2025-02-19

**Authors:** Sarah Kourouche, Julie Considine, Ling Li, Margaret Murphy, Ramon Z. Shaban, Mary K. Lam, Saartje Berendsen Russell, Margaret Fry, Christina Aggar, Michael M. Dinh, Amith Shetty, Timothy Shaw, Radhika V. Seimon, Nanda R. Aryal, James A. Hughes, Wayne Varndell, Kate Curtis

**Affiliations:** ^1^ Faculty of Medicine and Health The University of Sydney Susan Wakil School of Nursing and Midwifery Camperdown New South Wales Australia; ^2^ School of Nursing and Midwifery and Centre for Quality and Patient Safety Research in the Institute for Health Transformation Deakin University Geelong Victoria Australia; ^3^ Centre for Quality and Patient Safety Research – Eastern Health Partnership, Box Hill, VIC, Australia Box Hill Victoria Australia; ^4^ Australian Institute of Health in Macquarie University Macquarie Park New South Wales Australia; ^5^ Western Sydney Local Health District North Parramatta New South Wales Australia; ^6^ Faculty of Medicine and Health, Sydney Infectious Diseases Institute The University of Sydney Camperdown New South Wales Australia; ^7^ Research and Education Network & District Executive Western Sydney Local Health District Westmead New South Wales Australia; ^8^ New South Wales Biocontainment Centre Western Sydney Local Health District Westmead New South Wales Australia; ^9^ School of Health and Biomedical Sciences RMIT University Melbourne Victoria Australia; ^10^ RPA Green Light Institute for Emergency Care, Sydney Local Health District Camperdown New South Wales Australia; ^11^ University of Technology Sydney Faculty of Health Ultimo New South Wales Australia; ^12^ Northern Sydney Local Health District St Leonards New South Wales Australia; ^13^ Northern NSW Local Health District Southern Cross University Lismore New South Wales Australia; ^14^ New South Wales Institute of Trauma and Injury Management Croydon New South Wales Australia; ^15^ Sydney Medical School The University of Sydney Sydney New South Wales Australia; ^16^ System Sustainability and Performance New South Wales Ministry of Health St Leonards New South Wales Australia; ^17^ Biomedical Informatics and Digital Health The University of Sydney Sydney New South Wales Australia; ^18^ Faculty of Medicine and Health The University of Sydney Sydney New South Wales Australia; ^19^ Australian Institute of Health Innovation Macquarie University Sydney New South Wales Australia; ^20^ Faculty of Health, Centre for Healthcare Transformation, School of Nursing Queensland University of Technology Brisbane Queensland Australia; ^21^ Prince of Wales Hospital Emergency Department Sydney New South Wales Australia; ^22^ Faculty of Health University of Technology Sydney Ultimo New South Wales Australia; ^23^ College of Emergency Nursing Australasia (CENA) Beaumaris Victoria Australia; ^24^ Emergency Services, Illawarra Shoalhaven Local Health District Wollongong Hospital Wollongong New South Wales Australia

**Keywords:** barriers, enablers, Implementation science, implementation strategies

## Abstract

**Aim:**

The aim of this study was to (i) identify barriers and enablers and (ii) inform mitigating or strengthening strategies for implementing nurse‐initiated care protocols at scale in emergency departments (EDs).

**Design:**

Embedded mixed methods.

**Methods:**

The study included four clusters with a total 29 EDs in NSW, Australia. Concurrent quantitative and qualitative data were collected via electronic nursing and medical staff surveys and analysed. Barriers and enablers to implementation were identified and mapped to the domains of the Theoretical Domains Framework (TDF). Selection of intervention functions and behaviour change techniques (BCTs) enabled development of implementation strategies.

**Results:**

In total, 847 responses from nursing and medical staff (43%) reported four enablers for use and implementation: (i) knowing or being able to learn to use simple nurse‐initiated care; (ii) protocols help staff remember care; (iii) carefully considered education programme with protected time to attend training; and (iv) benefits of nurse‐initiated care. Nine barriers were identified: (i) lack of knowledge; (ii) lack of skills to initiate complex care (paediatric patients, high‐risk medications and imaging); (iii) risk for inappropriate care from influence of cognitive bias on decision‐making; (iv) punitive re‐enforcement; (v) protocols that are too limited, complex or lack clarity; (vi) perceived lack of support from medical or management; (vii) perception that tasks are outside nursing role; (viii) concern nurse‐initiated care may increase the already high workload of medical and nursing staff; and (ix) context. The barriers and enablers were mapped to nine TDF domains, five intervention functions and 18 BCTs informing implementation using strategies, including an education programme, pre‐existing videos, audit and feedback, clinical champions and an implementation plan.

**Conclusion:**

A rigorous, systematic process generated a multifaceted implementation strategy for optimising nurse‐initiated care in rural, regional and metropolitan EDs.

**Implications:**

Staff wanted safe interventions that did not lead to increased workload. Staff also wanted support from management and medical teams. Common barriers included a lack of knowledge and skill in advanced practice. Clinicians and policymakers can consider these barriers and enablers globally when implementing in the ED and other high‐acuity areas. Successful strategies targeting barriers to advanced practice by emergency nurses can be addressed at the local, state and national levels.

**Impact:**

Implementation of new clinical practices in the ED is complex and presents challenges. Key barriers and enablers, including those related to initiating care and workloads in the ED were identified in this study. This research broadly impacts ED staff and policymakers globally.

**Reporting Method:**

Mixed Methods Reporting in Rehabilitation & Health Sciences (MMR‐RHS).

**Patient or Public Contribution:**

Site senior nurse researchers for each cluster worked closely with site stakeholders, including local consumer groups. Consumer councils were engaged at all the sites. Site visits by the research nurses have been an important strategy for discussing the study with key stakeholders.

**Trial Registration:**

Australian and New Zealand Clinical Trial: ACTRN12622001480774p

**Summary**

- What does this paper contribute to the wider global clinical community?
  ○ Nurse‐initiated care is effective against problems in emergency departments globally, and this paper provides insight into the barriers and enablers to their implementation.
  ○ This paper established barriers to, and enablers of, nurse‐initiated care for successful implementation in the complex context of emergency care.
  ○ Many barriers and enablers reported were common across sites in this study despite their diversity and differences in nurse experiences.

## 1 Introduction

The introduction of nurse‐initiated care in emergency departments (EDs) can mitigate patient delays and improve time to treatment using interventions initiated by registered nurses prior to physician review, such as analgesia, diagnostic imaging or pathology (Burgess and Kynoch 2017). The impact of nurse‐initiated emergency care is well documented globally, reducing time to pain relief (Douma et al. 2016; Varndell et al. 2018; Burgess et al. 2021; Pierik et al. 2016; Wong et al. 2007), imaging (Douma et al. 2016) and treatment (Bruce et al. 2015) and decreasing admission rates (Burgess et al. 2021; Brown et al. 2016), patient morbidity and mortality and ED length of stay (Douma et al. 2016). Furthermore, it is safe and acceptable to patients in adult and paediatric populations (Considine et al. 2019; Deforest and Thompson 2012; Long et al. 2017). ED overcrowding is a global problem that nurse‐initiated care can mitigate by reducing delays, improving access to earlier care and disposition and service delivery (Hwang et al. 2016). Despite the benefits of nurse‐initiated care, it has often been locally implemented with varying and limited protocols and guidelines often associated with delays to treatment. Despite the evidence supporting the widespread use of nurse‐initiated emergency care protocols, their use has lacked consistency and standardisation.

## 2 Background

Successful and sustained implementation of interventions in EDs is hindered by system and staff factors (Southerland et al. 2023), which can be mitigated by using implementation science and mixed‐method approaches (Curtis et al. 2024). Although successful implementation has been demonstrated with smaller, single‐site studies with simple interventions, it has been less successful in complex large implementation studies (McInnes et al. 2020; Oliveira et al. 2023).

In response to state‐wide delays in assessment and treatment, in 2024, the New South Wales (NSW) Ministry of Health, through the Agency for Clinical Innovation (ACI), planned to implement the state‐wide ‘Emergency Care Assessment and Treatment (ECAT)’ Program comprising nurse‐initiated care across 172 NSW EDs. The programme includes 73 protocols (41 adult and 32 paediatric) to support and guide nurse‐initiated care in all EDs state‐wide. At the time of data collection for this study, ECAT protocols were being developed with medical, nursing, radiology, pathology, allied health and pharmacy stakeholders, and are now live across NSW EDs (https://aci.health.nsw.gov.au/ecat/). NSW is a large geographical state with metropolitan, rural and remote settings allowing for diverse settings to determine what support nursing staff anticipated they would need to implement the protocols.

International peer‐reviewed literature highlights the importance and opportunities to use behaviour change theory and implementation science to identify barriers and enablers to implementing nurse‐initiated emergency care in metropolitan, regional and rural EDs to yield data‐driven implementation strategies for optimising success (Guerin et al. 2022). This study was guided by the Behaviour Change Wheel (BCW), an implementation science model that synthesises 19 models of behaviour change that link the drivers of behaviour (capability, opportunity and motivation) to appropriate interventions and strategies (Michie et al. 2014). The Theoretical Domains Framework (TDF) is a framework that aligns with the BCW and is a synthesis of 33 behavioural change theories into 14 domains enabling comprehensive investigation of barriers and enablers to change (Cane et al. 2012).

## 3 The Study Aim

This study aimed to (i) identify barriers and enablers and (ii) inform mitigating or strengthening strategies for implementing nurse‐initiated emergency care protocols at scale in EDs.

## 4 Methods/Methodology

**FIGURE 1:**
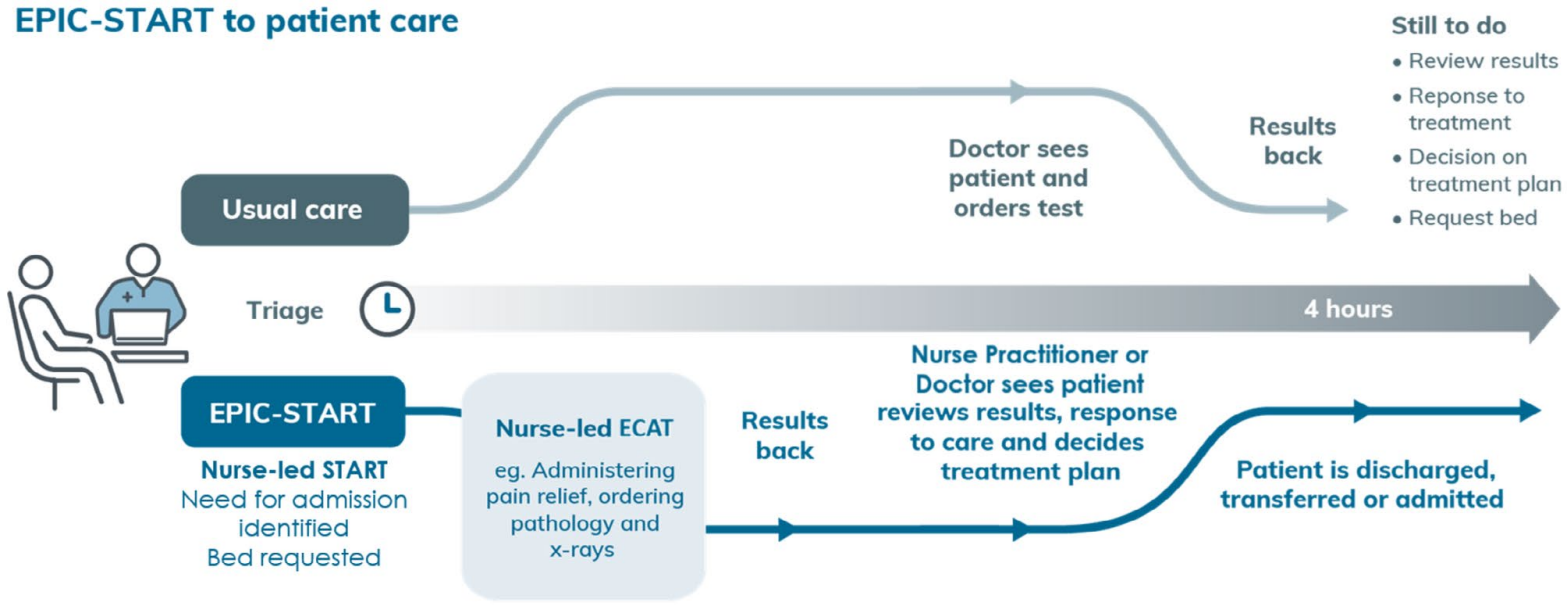
EPIC‐START model of care compared with standard emergency care. This figure demonstrates the difference in the usual pathway of care compared with the EPIC_START model of care. With the new model of care, patients receive nurse‐initiated investigations, diagnostics and treatments, and bed requests are made earlier, resulting in earlier disposition times. ECAT, Emergency Care Assessment and Treatment; EPIC, Emergency Protocols Initiating Care; START, The Sydney Triage to Admission Risk Tool. [Colour figure can be viewed at wileyonlinelibrary.com]

**FIGURE 2:**
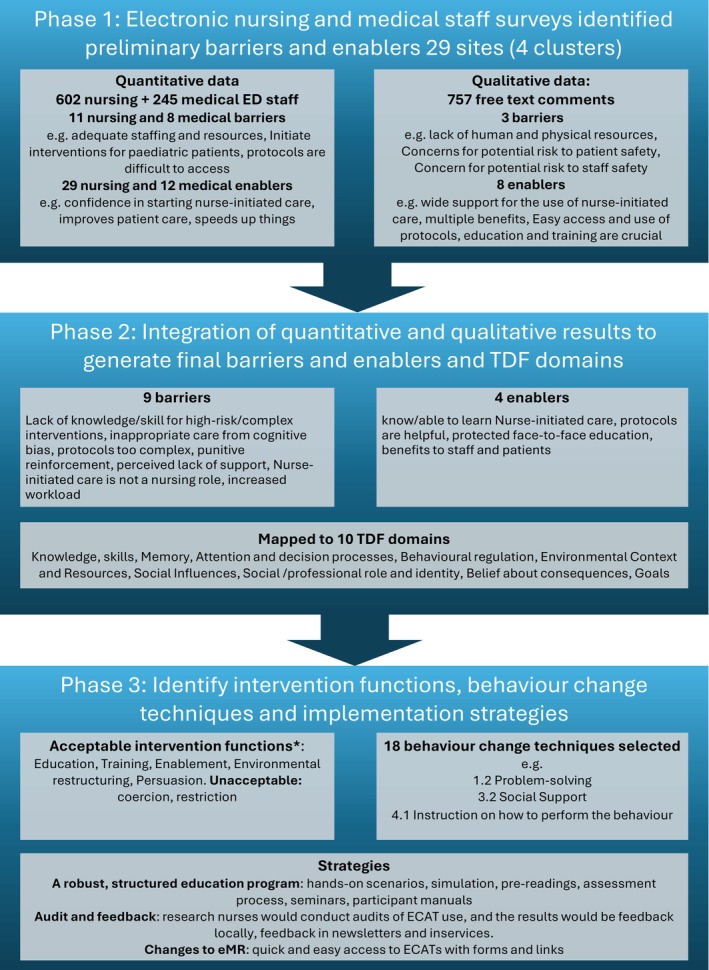
Flowchart summary of methods and results for identification of barriers and enablers and strategies. This figure demonstrates the three phases of the study and the associated findings at each phase leading to the key findings. *APEASE assessment, Affordability, Practicality, Effectiveness and cost‐effectiveness, Acceptability, Side‐effects/safety, and Equity; ECAT, Emergency Care Assessment and Treatment; ED, Emergency Department; e.g., example, eMR, Electronic Medical Records; TDF. Theoretical Domains Framework. [Colour figure can be viewed at wileyonlinelibrary.com]

This embedded mixed methods study (Creswell and Piano Clark 2011) forms the first phase of a multicentre hybrid implementation evaluation trial (Type II) (Wolfenden et al. 2021) called EPIC‐START (Curtis et al. 2023) (Figure 1). In this study, a comprehensive implementation strategy was needed for a complex intervention (73 nurse‐initiated care protocols) across multiple sites to optimise intervention uptake. To support implementation, it was first necessary to identify barriers and enablers of nurse‐initiated care using electronic surveys of emergency nursing and medical staff. This study was conducted in three phases to inform nurse‐initiated care protocol implementation (Figure 2) guided by the BCW. First, concurrent quantitative and qualitative data were collected via an electronic survey of ED nursing and medical staff and analysed separately to identify preliminary barriers and enablers. Second, integration of findings generated final barriers and enablers to nurse‐initiated care protocol implementation. Third, in a multi‐step process using the BCW, implementation strategies were identified that would be most effective in supporting behaviour change in nurses to use the nurse‐initiated care protocols and medical and nurse practitioners to endorse nurse‐initiated care (Michie et al. 2013). The state‐wide developed protocols (ECAT) had not yet been released and were not available for review in the survey; however, staff were asked to respond on the basis of their previous experiences with nurse‐initiated care in their context and what support they anticipated they would need.

### 4.1 Study Setting and Clusters

Four clusters with 29 EDs varying in size, resources and locations in NSW, Australia, were included in the study (Table 1). Clusters were based on geography and existing organisational structures called Local Health Districts. Resources at sites varied; rural EDs had limited or no on‐site medical, radiology and pathology resources; regional EDs had on‐site medical, radiology and pathology resources; and major city EDs had comprehensive services. Pre‐implementation site visits and steering committees were conducted to engage key stakeholders and inform survey distribution methods.

**TABLE 1:** Sites and contexts in EPIC‐START study.

Cluster (sites)	Description of LHD geography, population and site location and level^a^	ED patient presentations per year (2021–2022)	ED nurse numbers^b^	ED doctor numbers^b^
Cluster 1 (10EDs)	Spans 44,534km^2^ 200,000+ residents Location: 1 major city, 7 inner regional, 2 rural sites Level: 8 low, 2 mid‐high	111,637	180	48
Cluster 2 (12 EDs)	Spans 20,732km^2^ 300,000+ residents 1 major city, 9 inner regional, 2 rural sites Level: 9 low, 3 mid‐high	198,745	430	227
Cluster 3 (3 EDs)	Location: All sites in major city Level: 3 mid‐high	168,914	402	170
Cluster 4 (4 EDs)	All sites in major city Level: 4 mid‐high	201,390	280	217
Total 29 EDs		680,686	1292	662

Abbreviations: ED = emergency department, LHD = local health district.

^a^ Levels: low‐level: basic resources, may or may not have medical staff on‐site, no or limited radiology on‐site; mid‐high level: Can provide definitive care on‐site with comprehensive services, including intensive care, specialty services and allied health.

^b^ Numbers are estimates as staff are often transient.

### 4.2 Phase 1: Electronic Nursing and Medical Staff Surveys to Identify Preliminary Barriers and Enablers

#### 4.2.1 Sampling, Recruitment and Process

All nursing (*n* = 1294) and medical (*n* = 662) staff permanently employed in any of the 29 study EDs were invited to participate in an electronic survey via email by their nurse managers, medical directors or study research nurses. Surveys were administered via REDCap (Research Electronic Data Capture), a secure web‐based data management and survey delivery application. Advertisements with QR codes linked to the electronic survey were placed in staff meal rooms to remind participants and encourage participation. Participants were offered a $20AUD voucher on completion of the survey as an incentive and token for their time. Reminder emails were sent every 2 weeks to improve participation numbers. Participants consented electronically through REDCap and responses were anonymous. Participants were asked to enter their staff identification number (not linked to their name) to prevent multiple participation.

#### 4.2.2 Instruments With Validity and Reliability/Data Source

There were two electronic surveys: a 97‐item survey for emergency nursing staff and a 29‐item survey for emergency medical staff (Appendix S1). The surveys included open‐ended questions with closed questions enabling elaboration and capture of potentially missed perspectives and barriers. The surveys were adapted from similar questionnaires developed using the TDF (Cane et al. 2012; Swiger et al. 2017) and other validated change management tools (Swiger et al. 2017). All 14 TDF domains were assessed, including *Knowledge, Skills, Reinforcement, Memory, Social Influences, Environmental Context/Resources, Beliefs about Consequences, Optimism, Goals, Social/Professional role and identity, Beliefs about capabilities, Intentions, Emotion and Behavioural regulation* (Cane et al. 2012).

The nursing survey comprised five sections (Appendix S1) to address different aspects of nurse‐initiated care: (i) participant characteristics, (ii) perceptions of nurse‐initiated care in the department, (iii) confidence in nurse‐initiated care interventions, (iv) Practice Environment Scale of the Nursing Work Index (PES‐NWI) (Swiger et al. 2017) and (v) behavioural diagnostic questions based on the TDF to assess barriers and enablers. Five free‐text questions were used for elaboration and general comments on what staff members liked or disliked about nurse‐initiated care and what would help or hinder implementation at their site.

The medical survey included four sections (Appendix S1): (i) Participant demographics, including role, years of general experience and emergency medicine experience; (ii) perceptions of standardised nurse access to protocols and rationales; (iii) satisfaction with nurse‐initiated care; and (iv) the transition to new nurse‐initiated care protocols and potential impact of nurse‐initiated care protocol implementation on medical staff (Cane et al. 2012; Swiger et al. 2017). There were four open‐ended questions.

An advisory group of emergency nursing and medicine researchers and emergency nursing and medical staff with content expertise assessed both surveys for face and content validity. Items were phrased both positively and negatively to counter response set bias. The advisory group completed the survey on desktop computers and mobile devices, providing feedback on the functionality of the survey, completion time, typographical errors, questions irrelevant to their allocated role and the clarity of the instructions. Comments were collated, and only minor editorial changes were made to the survey on the basis of feedback.

### 4.3 Phase 2: Integration of Quantitative and Qualitative Results to Generate Final Barriers and Enablers and TDF Domains

Phase 1 findings were integrated using a rigorous process of consolidation, synthesis and comparison to derive the final barriers and enablers to nurse‐initiated care. The final barriers and enablers were then mapped to the TDF (Cane et al. 2012). Items that crossed multiple domains were allocated to the most relevant domain on the basis of the TDF definitions by one author (SK) and checked by a second author (KC). Discrepancies were dealt with using discussion to reach consensus among the research team.

### 4.4 Phase 3: Identify Intervention Functions, Behaviour Change Techniques and Implementation Strategies

The TDF domains were then mapped to intervention functions and behaviour change techniques (BCTs) likely to be most effective in addressing barriers and optimising intervention uptake (Michie et al. 2014). Intervention functions as guided by the BCW are broad categories designed to change the capability, opportunity or motivation to engage in behaviours (Michie et al. 2014). The BCTs are components that aim to alter behaviour and were selected from 93 techniques in the Behaviour Change Technique Taxonomy (BCTTv1) (Michie et al. 2013). The intervention functions and BCTs were each assessed for relevancy to the local context with the APEASE criteria (Affordable, Practical, Effective and cost‐effective, Acceptable, had Side‐effects and were safe and Equitable) (Michie et al. 2014).

Finally, the resulting BCTs were collated and operationalised using implementation strategies (modes of delivery) specific to the nurse‐initiated care protocols and context of the sites using information obtained from the survey. Representatives from each stakeholder group were consulted regularly throughout the implementation development process for agreement, for example, through a standing agenda item at cluster steering committee meetings (Michie et al. 2014).

### 4.5 Data Analysis

Quantitative data were analysed by statisticians in the investigator team (LL, NRA) using the Statistical Analysis System (SAS 9.4) (SAS Institute Inc., North Carolina, USA). Participants' characteristics were summarised using descriptive statistics. Categorical variables were presented as number and percentage [n (%)]. Mean and standard deviation (SD) were presented for normally distributed numerical variables and median and interquartile range (IQR) were presented for not normally distributed numerical variables. If any measures were missing, the percentages for categorical variables and summary statistics for numerical variables were calculated on the basis of the available values. Scales from 0 to 10 were categorised into three groups: disagree score of 0 to 4, neutral score of 5, scores 6 to 10 agree. Enablers with greater than 80% agreement or mean greater or equal to 8 to a positively worded statement in these groups. Barriers were defined as less than 80% agreement with a statement or mean of 8 or greater with a positively worded statement (Murphy et al. 2019). The PES‐NWI subscale score was evaluated on each subscale: barriers were defined as a score less than 3.0, and enablers were defined as scores equal to or greater than 3.0; scores of three and above are considered an excellent workplace index (Swiger et al. 2017).

Comparison analyses were performed using chi‐squared test or Fisher's exact test for categorical variables, *F*‐test of one‐way ANOVA for normally distributed continuous variables, and Kruskal–Wallis test for not normally distributed continuous variables. Fisher's exact test was used when low expected frequencies were present in cells of the contingency table. Two‐sided tests were conducted with a significance level of 0.05. Differences between clusters were noted.

Qualitative data from free‐text responses were imported into NVivo v14 (QSR International, Burlington, MA) and analysed collectively using the Graneheim (Graneheim et al. 2017) method of inductive content analysis. Analysis was by first author with expertise in emergency nursing but did not work at the sites. Analysis was checked by a second author (KC). The text was read several times to obtain a sense of the whole; the content was divided into meaningful units (codes) for analysis. The codes were abstracted to subcategories and categories at the highest level. This process was completed by one author (SK); increasing the reliability of the interpretation by limiting the number of coders (Douma et al. 2016). Any queries were resolved through discussion among authors (SK, KC). Categories derived from content analysis were considered barriers or enablers on the basis of positive or negative intonation and agreement by a second author. Quotes have been edited for simple spelling and grammatical errors.

### 4.6 Ethical Considerations

Ethics approval to conduct the trial was granted through the Sydney Local Health District Human Research Ethics Committee (2022/ETH01940) on 14 December 2022.

## 5 Results

Surveys were completed by 847 staff, with 602 (46.6%) and 245 (37.0%) response rates for nursing and medical staff, respectively. Characteristics varied across clusters in both surveys (Table 2). In the nursing survey, years of emergency and overall nursing experience were higher for rural/regional clusters. The medical respondents' emergency experience was highest at Cluster1 (only senior medical staff respondents) and Cluster3 (Table 2).

**TABLE 2:** Characteristics of participants in the nursing and medical surveys.

	ALL	Cluster1	Cluster2	Cluster3	Cluster4	*p* ^a^
Staff total *n* (%)	**847**	**140**	**247**	**204**	**256**	
Nursing role *n* (%)	602	109	187	125	181	
Registered nurse	465 (77.2%)	74 (67.9%)	142 (75.9%)	94 (75.2%)	155 (85.6%)	**0.015**
Enrolled nurse	20 (3.3%)	3 (2.8%)	8 (4.3%)	6 (4.8%)	3 (1.7%)	
Leadership role (e.g., nurse manager and nurse educator)	117 (19.4%)	32 (29.4%)	37 (19.8%)	25 (20.0%)	23 (12.7%)	
Medical role *n* (%)	245	31	60	79	75	
Emergency specialist	78 (31.8%)	14 (45.2%)	17 (28.3%)	28 (35.4%)	19 (25.3%)	**< 0.001**
Registrar	101 (41.2%)	17 (54.8%)	19 (31.7%)	38 (48.1%)	27 (36.0%)	
Junior medical	66 (26.7%)	0 (0.0%)	24 (40.0%)	13 (16.5%)	29 (38.7%)	
Years experience median [IQR]						
Nursing	7.0 (4.0, 16.0)	8.0 (5.0, 21.0)	10.0 (5.0, 21.0)	6.0 (4.0, 13.0)	5.0 (3.0, 8.0)	**< 0.001**
Medical	9.0 (4.0, 17.0)	12.0 (7.0, 22.0)	8.0 (4.0, 21.5)	9.0 (5.0, 20.0)	5.0 (2.0, 11.0)	**< 0.001**
Years emergency experience median [IQR]						
Nursing	4.0 (1.5, 10.0)	5.0 (2.0, 11.0)	5.0 (2.0, 15.0)	3.0 (1.0, 8.0)	3.0 (1.0, 6.0)	**< 0.001**
Medical	5.0 (2.0, 14.0)	10.0 (5.0, 20.0)	4.0 (1.0, 17.0)	7.0 (3.0, 17.0)	3.0 (1.0, 9.0)	**< 0.001**
Qualifications nursing *n* (%)						
Bachelor	237 (39.4%)	35 (32.1%)	37 (19.8%)	43 (34.4%)	122 (67.4%)	**< 0.001**
Certificate/diploma	302 (50.2%)	65 (59.6%)	131 (70.1%)	58 (46.4%)	48 (26.5%)	
Masters or higher	63 (10.5%)	9 (8.3%)	19 (10.2%)	24 (19.2%)	11 (6.1%)	

^a^ Chi‐squared test or Fisher's exact test for categorical variables and Kruskal–Wallis test for continuous variables, statistical significant in bold.

Detailed Phase 1 pre‐integration quantitative results are available in Appendix S2. Qualitative findings were generated from 547 nursing and 210 medical staff respondents who made free‐text comments. Comments were coded to 1126 nursing and 850 medical reference codes, then grouped into 10 subcategories and five overarching categories. The categories and subcategories with sample quotes are summarised in Appendix S2. The most prominent categories were staff being supportive of nurse‐initiated care protocols because of the considerable benefits to patients and staff and that successful and safe use of an expanded and standardised series of nurse‐initiated care will need robust, face‐to‐face clinician‐focused education and training in protected non‐clinical time (Appendix S2).

Four enablers and nine barriers to implementation mapped to nine TDF domains were used to develop strategies (Figure 2, Table 3). The barriers, enablers and TDF domains resulted in five intervention functions and 18 BCTs informing implementation using strategies, including an education programme, pre‐existing videos, audit and feedback, clinical champions and an implementation plan (Table 3).

**TABLE 3:** Barriers and enablers to nurse‐initiated care in the ED, mapped to theoretical domains framework, Intervention functions, BCTs and modes of delivery. [Colour table can be viewed at wileyonlinelibrary.com]

Theoretical domains (*and definition*)	Integrated barriers (B) and enablers (E)	Evidence—results from surveys, including quantitative and categories/subcategories from qualitative and *example quotes*	Intervention function (IF), *behaviour change techniques*, modes of delivery (in bold) and mechanism/content
Capability			
**Knowledge:** *An awareness of the existence of something*	Lack of knowledge to initiate radiology and high‐risk medications and paediatric interventions (B)	Low nurse confidence to initiate radiology investigations 6.0 (3.0, 9.0), thromboembolic medications 4.0 (0, 7.0) and interventions and for paediatric patients 6.0 (3.0, 9.0) (median [IQR]) Nurses currently accredited to initiate nurse‐initiated X‐ray low 159 (27.9%), Metro Clusters bigger barrier (*p* < 0.001) **Comprehensive clinician‐focused education and training is crucial to implementation (*n* = 335)** *With X‐rays there needs to be a good understanding of what to order and when to prevent the need for add‐ons or unnecessary X‐rays being done*—NSNW MO91	**Education (IF)** *BCT 4.1 Instruction on how to perform behaviour* *BCT 5.1 Information about health consequences* **Education programmes** to support existing knowledge and fill gaps. Instruction for more complex assessments and interventions, for example, assessment techniques for limb injuries, ordering protocols. Information on the health consequences and evidence for nurse‐initiated care **Videos** on nurse‐initiated X‐ray assessment and ordering
	Know or able to learn to use simple nurse‐initiated care (E)	Nurse confidence to initiate simple analgesia using nurse‐initiated protocols eg paracetamol, median (IQR) 10.0 (8, 10), antiemetics 8.0 (6.0, 10.0), pathology 8.0 (6.0, 10.0) I am interested in learning something new, 84.6% of nurses agreed **There is wide support for the use of NIP (*n* = 258)** *I work with a motivated nursing team who are proactive with initiating patient diagnostic and therapeutic care. Scope of practice at present is limited to analgesia, antiemetics, IVF [intravenous fluids] for haemodynamic resuscitation, they do identify sepsis and high risk chest pain re ACS and flag to MO for abi [antibiotics] to be charted—I do think they are capable of initiating abi [antibiotics] if a care pathway existed*—Cluster4 MO14	
**Skills:** *An ability or proficiency acquired through practice*	Lack of skills to initiate complex care (paediatric patients, high‐risk medications, radiology) (B)	Low nurse confidence to Initiate interventions for paediatric patients 6.0 (3.0, 9.0), radiology investigations—median 6.0 (3.0, 9.0), opioids (Cluster3 6.0 (3.0, 9.0)), thromboembolic 4.0 (0, 7.0), using nurse‐initiated protocols. Lowest at Cluster3 1.0 (0.0, 5.0) **Concerns for potential risk to patient safety** (*n* = 280) *Generally if something is deemed urgent enough to require steroids or bronchodilators in a paediatric patient the nurse approaches the doctor first, which I think remains best not only for confirmation of diagnosis but for repeated viewing of any change in the patients condition pre‐medication/intervention*—Cluster3 MO88	**Training (IF)** *6.1 Demonstration of the behaviour* *8.1 Behavioural Practice/rehearsal* *2.2 Feedback on behaviour* **Education programme**—Structured programme with demonstrations of skills in interactive workshops. In interactive workshops, participants demonstrate assessment and commencement of ECAT protocols. Participants are given opportunity to practice required skills **Educational materials** **Video** provided with demonstration of skills **Audit and feedback** of staff completion and feedback to staff to encourage improvement
**Memory, attention and decision processes:** *The ability to retain information, focus selectively on aspects of the environment and choose between two or more alternatives*	Risk for inappropriate care from influence of cognitive bias on decision‐making (B)	**Concerns for potential risk to patient safety** (*n* = 280) Potential risks related to cognitive Bias and decision‐making (*n* = 21) *Most patients we see have multiple issues that will require attention. A diagnostic “label” from a protocol may sometimes hinder approaching a patient with a broader perspective of ongoing issues—*Cluster2 MO75	**Enablement (IF)** *1.2 Problem‐solving* **Education programme** includes hands‐on scenarios and case studies so staff can troubleshoot and problem‐solve using real scenarios **Persuasion (IF)** *13.2 Framing/reframing* **Education programme** Demonstrate to staff use of NIP within current workflows and how can be used to enable care **In‐services/workshops** for medical staff regarding the role and workflow and training nurses will receive. Reassure and inform medical staff of the additional training and governance for high‐risk medications and interventions
	Protocols help to remind and inform appropriate care (E)	It is easy to remember something new, 85.5% nurses agree Advancement of nursing as profession improving nursing care (*n* = 219): *…will be great tool to give chance to nurses to do things beyond their regular nursing scope of practice which will give positive effect on patient's care*—Cluster3 N114	
**Behavioural regulation:** *Anything aimed at managing or changing objectively observed or measured actions*	Punitive re‐enforcement is not helpful (B)	Knowing that change is being monitored, agree 64.4% Knowing there are consequences if we don't change, 54.5% agree Social supports needed (*n* = 170): *Knowing the change is being monitored and that there are consequences. Most nursing staff behind are hard working and have a work ethic would want to embrace the changes to see improvements in practice and won't need to be monitored or “watched”*—Cluster1 N67	**Enablement (IF)** *3.2 Social Support (practical)* *3.3 Social support (emotional)* **Clinical champions, research nurses** and CNEs to provide on‐site clinical support and reinforcement of behaviours, reviewing goals for use of protocols and if achieving goals
Opportunity			
**Environmental context and resources** *: Any circumstance of a person's situation or environment that discourages or encourages the development of skills and abilities, independence, social competence and adaptive behaviour*	Protocols that are too limited, complex or lack clarity hinder use (B)	Previous NIP were too limited, 216 (35.9%) nurses agree Interventions were too limited in existing protocols, Yes 216 (35.9%), SSNW highest 56.0% There needs to be more protocols, 65% agree, SSNW highest 87.2% Protocols that are too limited hinder use (*n* = 198) *The protocols are very limited. Pain relief is only panadol and most people have taken panadol before coming. They are vague and this sometimes can cause confusion*—Cluster1 N56 Protocols that lack clarity hinder use (*n* = 93) Easy access and use of protocols is essential for use (*n* = 180) *The current ones a sometimes hard to locate so it's easier to order things individually. Would still use the same orders within the protocol. But it would be nice if they are easy to locate*—Cluster3 N7	**Environmental restructuring (IF)** *12.1 Restructuring the physical environment* **ECAT protocols** developed through extensive state‐wide process led by NSW Health to standardise **eMR changes** to include links to ECAT protocols and easier ordering process

Protected time to attend a rigorous, evidence‐based in‐person education programme, will enable implementation (E)	Face‐to‐face education, mean 8.8 (1.7) There is enough time to change the way of working, 65.7% agree Allow flexibility, support and time in implementation without pressure (*n* = 111) *Enough time and resources for it to be safely imbedded in the department. Enough support and training for it to be done properly to give staff the confidence to put the leaning into practice …. Please don't rush it! Allow time for the roll out to be done properly. Its an important project and should be done right*—Cluster1 N54 **Successful and safe use of an expanded and standardised series of NIP will need robust, face‐to‐face clinician‐focused education and training in protected non‐clinical time (*n* = 641)** *Need more skills, these skills would come in handy when needed, especially during night shift when there's very limited medical respite*. I.e. *ABG, CXR, pelvic XR, suture, local anaesthetic, more nurse initiative meds*—Cluster4 N129 *Meaningful education sessions where we can interactively learn and try the system out not posters/emails/eLearning modules—these don't work*—Cluster3 N15	**Environmental restructuring (IF)** *12.2. Restructuring the social environment* **Education programme** Allow protected time off the floor for staff to attend training. Limit pre‐reading material **Ongoing training** for example in‐services to continue learning after the programme
**Social influences:** *Interpersonal processes that can cause individuals to change their thoughts, feelings or behaviours*	Perceived lack of support from medical teams and/or management will hinder implementation and ongoing use (B)	I feel supported by the medical team in using nurse‐initiated protocols (agree 66.5%) I feel supported by management (agree 67.2%) Social supports needed (*n* = 170): *Acknowledgment from the medical officers that they are aware of the new policies and protocols in place that can be attended by nurses to decrease any conflict between nurses and medical officers*—Cluster2 N128	**Enablement (IF)** *1.9. Commitment* *6.3 Information about others' approval* Demonstrated commitment from executive sponsors in local health district in writing Use advisory boards and workgroups eg local steering committee to support implementation and identify potential risks and issues Ensure management support is evident through education time, and change in practice Medical support visible in education programme **Education programme** Using train the trainer strategies with local clinical leaders attending as trainers
Motivation			
**Social/professional role and identity:** *A coherent set of behaviours and displayed personal qualities of an individual in a social or work setting*	Perception that nurse‐initiated care is not nursing role will hinder implementation (B)	Doctor: It is beyond the scope of nursing practice, 16.7% agree (Cluster1 25.8%) There is increased responsibility for nurses with nurse‐initiated care (*n* = 76) *I want to use NIP because it benefits the patients. I do not want to use them if it means increased workload and burden on the nurses to do everything*—Cluster2 N177	**Persuasion (IF)** *9.1 Credible source* *5.2 Salience of consequences* **In‐services/meetings** for medical staff on evidence for nurse‐initiated care delivered with support from medical leaders Meetings with pathology, radiology, pharmacy teams on change
**Belief about consequences:** *Acceptance of the truth, reality or validity about outcomes of a behaviour in a given situation*	Concern nurse‐initiated care may increase the already high workload of medical and nursing staff (B)	Medical: It would be easier for me to just do it myself 19.6% agree (Cluster1 38.7%) Medical: It will increase my administrative workload, 20.0% agree (Cluster1 29.0%) Medical: It will be more work for me later, 13.5% agree (Cluster1 32.3%) Staffing and resource adequacy Mean (SD) 2.39 (0.68) out of 4 A lack of human and physical resources hinders implementation (*n* = 162) *the floor is already busy so extra nurses are needed to successfully apply this approach. We already do not have enough time to apply basic clinical care even before stating EPIC*—Cluster3 N30	**Persuasion (IF)** *5.3 Information about social and environmental consequences* **In‐services/meetings** to provide information to medical staff on evidence of nurse‐initiated care, impact on emergency care

Implementation will fail if the practice environment is not considered (B)	Nursing: A single method will suit all situations in the ED (39% agree) Nursing: A standard approach will work (53.1% agree) Nursing: The training can be standardised (39.2% agree) Nursing: The training will not be time consuming (agree 66.3%) Change should consider the context of the sites (*n* = 46) and The experience of the nurse will impact the success of NIP (*n* = 81) *It needs to be focused on senior, experienced nurses who have reached a certain level of training and are able to demonstrate their competence. Too many new nurses fresh from uni coming into ED and it would not be appropriate for them to be initiating ED care*—Cluster2 N9	*1.4 Action planning* **Implementation plan** for each site, including who is making the changes, and timeframes and milestones, and progress measures **Tailor to context**—slide in education programme to show how has been tailored
**Goals:** *Mental representations of outcomes or end states that an individual wants to achieve*.	The multiple benefits of nurse‐initiated care to patients and staff align with clinician values (E)	Patients will appreciate my being able to give them treatment earlier, 89.1% agree Improves patient care, 83.7% (Cluster3 77.6%) There are multiple benefits to implementation (*n* = 849), Improved patient care and experience (*n* = 354). *Allows me to provide symptomatic relief to patients whilst they are waiting to be seen by medical staff*—Cluster4 N96	**Persuasion (IF)** *2.7 Feedback on outcome of behaviour* **Audit and feedback** to staff on impact ECAT having on patients and time to treatment to encourage continued use

*Note:* **Intervention functions (IF)—Education:** *increasing knowledge and understanding by informing, explaining, showing and correcting*; **Training:** *increasing psychological or physical skills, or habit strength by explanation, demonstration, practice, feedback and correction*; **Enablement:** *providing support to improve ability to change in a variety of ways not covered by other intervention functions*; **Environmental restructuring:** *constraining or promoting behaviour by shaping the physical or social environment*; ***Persuasion:** use communication to induce positive or negative feeling to stimulate action*.

### 5.1 Barriers and Enablers

The nine barriers and four enablers are grouped into the BCW categories of capability, opportunity and motivation (Table 3).

#### 5.1.1 Capability

Barriers related to the capability theme were lack of knowledge and skills to initiate complex care (paediatric patients, high‐risk medications and imaging), and risk for inappropriate care from the influence of cognitive bias on decision‐making. Enablers were knowing or being able to learn to use simple nurse‐initiated care and protocols that help staff remember care.

There was a lack of knowledge and skills to initiate radiology, high‐risk medications and paediatric interventions. These findings were consistent with low nurse confidence scores and low satisfaction ratings from medical respondents. For example, nurse confidence in initiating care was low for paediatric care 6.0 (3.0–9.0), radiology 6.0 (3.0 to 9.0) and thrombolytics 4.0 (0.0 to 7.0) (median (IQR)) (Appendix S2). Confidence rates were higher (*p* < 0.001) for most interventions in Clusters 1 and 2 where more experienced nurses were used to more autonomy. Medical respondents reported low satisfaction scores for interventions with paediatric patients (6.0 (5.0–8.0)) and thromboembolics (5.0 (0.0–6.0)). However, medical satisfaction with radiology was higher at 7.0 (5.0–8.0), which may be due to their experiences with experienced nurses. A large site in Cluster4 did not have paediatric presentations which may have influenced their lower scores. Qualitative comments supported these findings, urging the need for targeted skills and knowledge prior to implementation, especially for more inexperienced staff. Overwhelmingly, inadequate education was a barrier to implementing and using nurse‐initiated care, with the view expressed that nurse‐initiated care needs a robust, comprehensive, clinician‐focused, standardised educational programme. Furthermore, education was reported to be as essential to reduce risks:

As long as the nurses have been adequately trained and correctly choose the right patient to apply the protocol, then it will significantly improve patient care, decrease time etc. However, applying the protocol to the incorrect patient may, of course, be detrimental to care and increase workload. Therefore, much care must be taken to ensure that the correct training and education and support is applied to ensure the former is the case. (Cluster2 MO54)

In contrast, and unsurprisingly, staff reported high confidence in initiating more ‘simple’ interventions like analgesia (median 10.0 (8.0–10.0), antiemetics 8.0 (6.0–10.0) and pathology 8.0 (6.0–10.0)). This confidence was considered an enabler, ‘Know or able to learn to use simple nurse‐initiated care’ and demonstrates the importance of prior exposure.

Another reported barrier was the risk of inappropriate care from the influence of cognitive bias on decision‐making. Medical and nursing staff identified risks and raised considerations for training nursing staff in using the protocols safely and effectively. Of particular concern was the potential for errors if nursing staff commence an incorrect protocol, leading to cognitive bias for medical staff. Nursing respondents had similar concerns about the clinical experience nurses need for clinical judgement:

Requires advanced clinical judgement/critical thinking of the patient. (Cluster3 N77)

However, it was an enabler that *‘*Protocols help to remind and inform appropriate care’. Respondents described protocols as providing a framework for emergency nursing care supporting decision‐making for typical presentations.

It will lead to some inappropriate investigations which cost money and potentially prolong treatment time but it will significantly improve time to treatment, and junior doctors cause just as many issues with inappropriate investigations as protocolised nurses do. (Cluster2 MO55)

#### 5.1.2 Opportunity

Enablers of opportunity included education programmes with protected time to attend training, and barriers were protocols that were too limited, complex or lacked clarity, as well as a perceived lack of support from medical or management.

Respondents held that a thorough and robust education programme was needed to enable nurse‐initiated care protocol implementation and use, with only 53.1% of nurse respondents reporting that a standard implementation strategy would work. Respondents reported that there needed to be consideration of the nurses' time constraints and protected time to attend training (93.7%) and support in the clinical environment to adjust (93.9%). Furthermore, respondents reported that the training should consider the nurse's experience and the site's context, such as rural versus metropolitan, and the available supportive resources. Respondents reported that the education programme needs to be comprehensive and supported with protected education and training time, as described by this nurse:

TIME! Time for the nurses to attend to the training and to attend to the HETI [Health Education and Training] online modules and anything else that gets rolled out‐ not having education applied in an ad hoc way when the floor isn't busy. (Cluster2 N18)

Nurse respondents reported a ‘perceived lack of support from medical teams and management’ for the future implementation and ongoing use of the nurse‐initiated care protocols, with low nurse agreement on enough clinical support (62.9%), medical support (66.5%) or management support (67.2%). However, the medical survey findings refuted these perceptions—medical respondents indicated they strongly supported nurse‐initiated care (92.2% agree). Medical respondents did, however, stress the need for precautions to minimise risks and increase staff training. Some respondents also expressed concerns regarding the lack of support from senior management and other nursing staff. They felt it was vital to have the support of senior nursing staff, such as educators, for the transition.

Nursing respondents had many comments and concerns about how the protocols would be developed, stating that limited, complex or unclear protocols would hinder use. Nurse respondents agreed that there needed to be more protocols (65.0%), as an example from this nurse respondent:

[The previous protocols were] very restrictive range and hence don't really meet the needs of patients. It's well within a nurse's abilities to be using increased pharmacology provided clear guidelines and boundaries set. (Cluster1 N54)

Likewise, medical respondents expressed the need for extensive evidence and governance to support the protocols. Nursing respondents expressed that protocols must be clear and easy to follow and have a simple process to make initiation as easy as possible in a workplace with a high workload; as one respondent stated:

Make them easier to access and order. (Cluster1 N15)

#### 5.1.3 Motivation

The anticipated benefit of nurse‐initiated care was a significant motivating enabler. The barriers included perceptions that tasks are outside the nursing role, concerns that nurse‐initiated care may increase the already high workload of medical and nursing staff and the ED context.

Considerably, the implementation of nurse‐initiated care aligned with the goals and values of the staff surveyed. Nurse respondents felt the implementation of nurse‐initiated care protocols would increase patient appreciation for earlier treatment (89.1% agree), be easy to remember (85.5%) and improve patient care (83.7%) (Appendix S2). Medical respondents reported that the implementation of nurse‐initiated care protocols would lead to better patient care (89.4%), save time (88.6%), reduce time to treatment (85.7%) and reduce time to diagnosis (82.0%) (Appendix S2). Furthermore, clusters scored well on the nursing work index subscale for collegial nurse‐physician relationships. Nurse respondents expressed that implementation would benefit nurse autonomy and patient care and outcomes:

I like how it allows the nursing staff to give [the] patient appropriate care without chasing the doctor up and delaying care. Giving [the] patient immediate pain relief is extremely beneficial, creates a less hostile environment as patients can become extremely aggressive and rude when not receiving pain relief. (Cluster4 N153)

As did medical respondents:

I think it will be great to hone in on nurses' assessment skills and cater care accordingly. It will give nurses the ability to initiate care confidently as these protocols are obviously governed by evidence and hopefully supported by medical team. I think it will allow for faster care, and reduce delays that are caused waiting for medical staff to chart things. (Cluster3 MO142)

However, several respondents expressed apprehension that nurse‐initiated care may increase the already high workload of medical and nursing staff, especially when combined with inadequate training and support for the transition. One‐fifth (20%) of medical respondents reported that nursed‐initiated care would increase their administrative load, and similarly, 19.6% reported that it would be easier to do it themselves. Medical respondents from Cluster1 had the highest responses to administrative workload (29.0%) and also reported that nurse‐initiated care was beyond the scope of nursing practice (25.8%) and would be more work for them later (32.3%) which may explain the Cluster1 nurse high disagreement on medical support. Nurse respondents stated that a high workload would impact their ability to initiate care:

Often, I don't have time to commence them as the triage nurse, and there are more patients waiting to be triaged. The nurse in charge is often busy in resus or relieving breaks so sometimes does not have time to commence either. (Cluster1 N64)

Few nursing and medical respondents felt that initiating care is not a nurse's role. Some respondents preferred that nursing staff contact medical staff when in doubt about more complex patients, and others inferred that standard protocols would not meet local needs.

nurse‐initiated care is too limited to emergency presentations and doesn't take into consideration the GP type presentations in a small rural site where no doctor is available. There are protocols that have been working for more than 20 years in other states that cover these presentations and allow for a better service to the community. (Cluster2 N39)

A few of the qualitative comments suggested that initiating care was a medical role and that they were uncertain of the impact of nurse‐initiated care and increased remuneration of staff, for example:

Extra work for nursing staff when we are already so low on time to perform the tasks expected of us, which I believe can take away from the quality of patient care. I also believe that nurses will be participating in a doctor's role with assessing the need and charting of IV [intravenous] antibiotics and that will an increase in skill should come and increase in pay. (Cluster4 N109)

### 5.2 Implementation Strategies

The strategies selected to mitigate barriers and facilitate enablers included an education programme, pre‐existing videos, audit and feedback, clinical champions and an implementation plan for each cluster. Using the BCW, the final barriers and enablers and TDF domains from Phase 2 were mapped to five Intervention functions (Table 3): (i) education, (ii) training, (iii) enablement, (iv) environmental restructuring and (v) persuasion. These were subsequently mapped and assessed using the APEASE criteria resulting in 18 BCTs (Table 3).

A robust, structured education programme for face‐to‐face learning was identified as an important strategy to address barriers and enablers targeting multiple BCTs (Table 3). Nurses reported time to attend learning, opportunity to ask questions (93.7%) and face‐to‐face education (93.5%) as supportive of their successfully implementing nurse‐initiated care protocols (Appendix S2). This was supported by the qualitative findings, with in‐person, engaging, face‐to‐face education and training preferred over eLearning. Considerations for developing the education programme were evidence‐informed educational principles, intended learning outcomes, protected face‐to‐face learning, and development and delivery with highly experienced emergency nurses and educators.

The second strategy to address barriers was to use clinical champions to improve local clinical support and ongoing training. Clinical champions would provide this role as local clinical leaders. For example, staff from the sites could be allocated as instructors for the education programmes and to provide clinical support during implementation. Clinical champions can be volunteers or can be selected for their impact locally. Clinical champions would be given further resources from the research team to support their role.

Audit and feedback were identified as strategies to operationalise the BCT ‘feedback on behaviour’. Research nurses would conduct audits of nurse‐initiated care protocol use, and the results would be feedback locally using positive reinforcement using locally approved methods. Nursing respondents did not want their actions monitored punitively but to be supported by positive feedback and communication that respected them and recognised their value. Other strategies identified included changes to the medical record system, providing ongoing training, implementation plans for each site and local advisory groups.

## 6 Discussion

### 6.1 Key Findings

This study used an embedded mixed methods approach to identify the barriers and enablers to implementing nurse‐initiated care protocols and develop strategies to support their implementation in 29 EDs. The findings from the nursing and medical surveys yielded four enablers and nine barriers and enablers across capability, opportunity and motivation domains. In a rigorous process using the BCW, the barriers and enablers were then mapped to nine TDF domains, five intervention functions and 18 BCTs informing implementation using strategies, including an education programme, pre‐existing videos, audit and feedback, clinical champions and an implementation plan.

### 6.2 Barriers, Enablers and Strategies in the Literature

Nurses reported they lacked knowledge and skills for paediatric interventions, high‐risk medications and radiology initiation. Evidence for nurse‐initiated care, such as radiology, has been well‐established in the literature (Considine et al. 2019). However, nursing and medical staff reported low confidence or satisfaction with initiating imaging. Lack of knowledge and skills is not surprising for advanced practice; however, prior exposure and experience can help implementation as demonstrated in work from Canada (Jabbour et al. 2018), supporting the use of a targeted education programme that considers nurse experience. Organisational factors, such as workload and social supports, including manager and leader influences, could impact implementation and are often concerns for interventions with emergency nurses (Clement et al. 2016). For example, in a study on pain management in the ED from the United States, workload and process of documentation were barriers, identifying concerns that nurse‐initiated analgesia may impact already busy workflow and interrupt other tasks (Burgess et al. 2024). Risk around nurse‐initiated care protocols was identified as a risk; however, this finding was limited and almost always accompanied by the suggestion of including education, training or support to mitigate the risk.

Enablers are also important to consider for strengthening strategies. In a recent Australian single‐site study on nurse‐initiated care (Gawthorne et al. 2025), enablers were equally related to the benefits of nurse‐initiated care on patient outcomes and departments, supporting the need for strategies that provide proof of evidence and alignment with staff goals. The access and design of nurse‐initiated care protocols were reported as imperative for the implementation of nurse‐initiated protocols in this study, with innovation designs often having major impact on real‐world implementation efforts as identified in a US study on barriers in real‐world settings (Dir et al. 2021). Furthermore, a key enabler was quick and easy access to the nurse‐initiated care protocols through the electronic medical record. Strategies to support this enabler were planned at a state level, and some would need to be addressed locally.

### 6.3 Strengths and Limitations

In this study, we focused on the clusters for implementation, as this was necessary to inform the implementation of the nurse‐initiated care protocols at study sites, as the first phase of a larger study a cluster randomised controlled trial in progress across 29 NSW EDs (Curtis et al. 2023). It was not within the scope of this paper to report on differences between geographical areas and all hospital resources. However, further research may investigate the commonalities and differences between locations.

A limitation of the study was that the state ECAT protocols were unavailable to ED staff when the surveys were conducted, which may have influenced their responses. Without access to the final protocols, respondents could not fully assess or engage with the content of the ECAT in practice. As a result, their feedback on protocol design, clarity and usability was based on perceptions of pre‐existing protocols or general concerns about protocol implementation, rather than a direct evaluation of the ECAT protocols themselves. We did not collect participant gender in the surveys as a standard demographic variable, which may impact the generalisability of the results; however, as gender would not be considered in strategies, it was considered unnecessary.

Some other typical factors limit this study. Although our response rates give us confidence in a good level of representation from frontline clinicians, there may have been an over‐representation among participants of leaders and educators. Participation was voluntary, and those who agreed may have been early adopters or were otherwise more motivated. As such, our findings may be an optimistic perspective of the willing. We may not have heard sufficiently from those more constrained by challenges in the clinical setting or unwilling to undergo practice changes.

### 6.4 Recommendations for Further Research

This paper fills a significant research‐practice gap in its use of implementation theories to understand clinical practice change in a complex and dynamic ED environment with nurse‐initiated care. There is currently a paucity of implementation‐based research evidence in ED settings and with advanced practice innovations. A strength of this study was that it was a rigorous process that used the implementation science frameworks BCW and TDF. However, which framework is most appropriate for the review of barriers at scale is less certain (Guerin et al. 2022). Although some frameworks like the Consolidated Framework for Implementation Research (CFIR), include organisational factors, the use of the BCW and TDF was adequate for this study, with organisation factors covered in the ‘environment’ domain in a less complicated approach (Damschroder et al. 2022).

The barriers and enablers identified in this study are important to the success of the implementation strategy within a complex clinical setting. The factors identified will also help articulate key steps and assess fidelity post‐implementation. These findings will help guide further ED implementation research, adding to the body of implementation evidence in this unique setting. More research is needed to compare barriers and enablers in different settings to help inform implementation efforts. Nurse‐initiated care is known to have many benefits locally; implementation at scale will allow further understanding of the more widespread impacts, such as ED overcrowding and healthcare workforce shortages.

### 6.5 Implications for Policy and Practice

Implementation in EDs is complex and a detailed understanding of the barriers and enablers to change in this context is essential. Health organisations and policymakers must use evidence‐informed strategies to support implementation efforts, such as by using the TDF and BCW, to guide intervention design as was done in this study. Common enablers and barriers from this study may be used to help inform implementation plans globally. For example, this research demonstrated that emergency staff wanted safe interventions that did not lead to increased workload. Staff also wanted support from management and medical teams. Clinicians and policymakers can consider these barriers and enablers when implementing in the ED and other high‐acuity areas. Policy changes at state and national levels may assess and address organisational barriers to acknowledging the advanced practice of the emergency nurse, especially the need for protected time for learning and adaptation and necessary resources.

## 7 Conclusion

Detailed barriers and enablers to implementing nurse‐initiated care within an ED setting have been identified by frontline emergency nursing and medical staff. Emergency nursing and medical staff reported strong support for nurse‐initiated care given perceived benefits for patients and nurse advancement. By using the TDF to explore these factors and the BCW to conceptualise the barriers and enablers that might interact to effect change the study has ensured a systematic approach. Tailored implementation strategies that consider the unique local needs and challenges, such as addressing educational needs and potential safety risks, are crucial for fostering a supportive environment for protocol use and intervention uptake. In this study, barriers and enablers across diverse settings and nurses' experiences were identified and underpinned by sophisticated solutions for implementation success that are relevant to other emergency contexts globally. Data‐driven, evidence‐informed implementation strategies to maximise optimal uptake of the nurse‐initiated care protocols were developed as part of a larger clinical trial drawing on international implementation science evidence for standardisation at scale.

## Author Contributions

K.C., M.D., R.Z.S., A.S., M.F. and J.C.: conceptualisation and design of the study. L.L. and M.K.L.: statistical design. T.S., M.M., C.A., S.B.R., R.V.S., K.A.H., J.A.H., S.K. and W.V.: further refinements to the design. S.K., L.L., K.C. and N.R.A.: data curation, formal analysis. K.C., M.D., R.Z.S., A.S., M.F., J.C., S.K. and C.A.: funding acquisition. S.K., L.L., M.M., S.B.R. and N.R.A.: investigation. K.C., M.D., R.Z.S., A.S., M.F., J.C., L.L. and M.K.L.: methodology. S.K., R.V.S., C.A. and M.M.: project administration. K.C., R.Z.S., J.C., L.L. and M.D.: supervision. S.K., L.L., K.C. and N.R.A.: validation, visualisation. S.K.: writing – original draft of the paper with significant input from K.C. All authors contributed to the writing – review and editing, contributed substantially to the conception and design of the work and drafted or substantively revised the work, approved the submitted version and agreed both to be personally accountable for their contribution and ensure that questions related to the accuracy or integrity of any part of the work.

## Ethics Statement

The Sydney Local Health District Research Ethics Committee (reference number: 2022/ETH01940) provided ethical approval on 14 December 2022.

## Conflicts of Interest

The authors declare no conflicts of interest.

## Statistical Analysis

The authors have checked to ensure our submission conforms as applicable to the Journal's statistical guidelines. There are statisticians on the author team—L.L., N.R.A. and M.K.L.

## Supporting information


Appendix S1.

Appendix S2.

Appendix S3.

## Data Availability

The data that support the findings of this study are available on request from the corresponding author. The data are not publicly available due to privacy or ethical restrictions.
