# Supplementary material for: Identifying Barriers and Enablers for Nurse‐Initiated Care for Designing Implementation at Scale in Australian Emergency Departments: A Mixed Methods Study
Source: J Clin Nurs. 2025 Feb 19;34(7):2718–36. doi: 10.1111/jocn.17693 (PMC12181148; doi:10.1111/jocn.17693)
Supplement: Supplementary file 1 — Appendix S1. [file JOCN-34-2718-s001.docx]

# Surveys

# NURSING SURVEY

*The survey was managed in REDCap. The content of the online survey is below.*

| **Instrument** | **Items** | **Description** |
| --- | --- | --- |
| Participant characteristics | 6 | qualifications, years of general and emergency nursing experience, areas worked, and current clinical accreditations (e.g. nurse-initiated medications, nurse-initiated x-ray and local nurse-initiated standing orders) were sought |
| Nurse-initiated care | 23 | to establish barriers to and enablers of existing nurse-initiated protocols from 0 (Strongly disagree) to 10 (Strongly agree). |
| Self-confidence | 12 | establish current confidence in nurse-initiated care interventions, e.g. pathology, radiology, and bronchodilators, from 0 (no confidence) to 10 (complete confidence). |
| Practice Environment Scale of the Nursing Work Index | 27 (5 sub-scales) | a validated 27-item and five subscale tool using a 4-point Likert scale to determine organisational and other factors that influence a nurse’s ability to practice nursing skilfully and deliver high-quality care [24] rated from 1 (Strongly disagree) to 4 (Strongly agree). |
| Behavioural diagnostics | 29 | based on the TDF and asked respondents to score factors likely to help or hinder nurse-initiated care protocol implementation from 0 (Strongly disagree) to 10 (Strongly agree). |

## Instrument 1: Characteristics

1. **Current position** (Select one):

- RN
- EEN
- Leadership role - NUM/ CNC/ CNS/ NP / CNE
- other (specify) ____________

1. **How many years have you worked as nurse?** Years (0-70max)
2. How many years have you worked in the **emergency department**? Years (0-70max)
3. **Please tick your highest level of post graduate nursing qualification:**

- None
- Graduate Certificate
- Graduate Diploma
- Masters or higher

1. **What areas of the ED do you work?**

(Select all that apply)

- General acute area
- Fast track
- Urgent Care
- Paediatrics
- Triage
- Resuscitation room
- Clinical Initiatives Nurse
- Emergency short stay area / unit
- Advanced Practice nurse
- Other __________________________

1. **Please select the facility(ies) that you currently work at:**

*(Will provide selection options for all sites within the LHD participating in the study)*

1. **Which of the following accreditations do you have?** (select all that apply)

- Transition to emergency Practice
- First Line Emergency Care Course (FLECC)
- HIRAID (History, Identify Red flags, Assessment, Interventions, Diagnostics
- DETECT (Detecting Deterioration, Evaluation, Treatment, Escalation and Communicating in Teams)
- Rural Nursing Pathways in Practice (RNPIP)
- Nurse Delegated Emergency Care (NDEC)
- Advanced Cardiac Life Support (ACLS)
- Resus for kids
- Suturing
- Plastering
- Other _________________________

## Instrument 2: Nurse-initiated care

Nurse-initiated care can be defined as nursing staff autonomously providing care to patients prior to medical review. Examples are administering analgesia, bronchodilators, imaging and pathology requests.

1. Do you have nurse-initiated processes in your department?

- Yes
- No
- Unsure

IF YES go to part A, if no or unsure go to Part B

**Part A**

1. Are you accredited in your department to provide nurse-initiated care (ED approved protocols)?

- FLECC
- Nurse-initiateded medications (NIM)
- Nurse initiated xray (NIX)
- Nurse Delegated Emergency Care (NDEC)
- Department specific
- Other _________________

1. **What do you like about nurse-initiated processes in your department?** (check all that apply)

- Ease of access of protocols/ guidelines
- Clear protocols/ guidelines for use
- Speeds things up for my patient
- Easy to follow Protocols/ guidelines
- Improves patient care
- Medical staff appreciate it
- Patient appreciates it
- Autonomy
- Professional development / satisfaction
- Make a difference to patient care
- Acknowledges or validates my practice
- Other _______

Anything else? (free text)

1. **What don’t you like about your current nurse-initiated protocols?** (Select all that apply)

- Difficult to access
- Guideline too complex
- Guideline too vague
- Too many steps involved
- Does not help patient
- Interventions are too limited
- Medical staff don’t support
- Other _______

Please elaborate on how you would like them improved: (free text)

## Instrument 3: Self-confidence in nurse-initiated care

Read the statement below and circle the number to the right which indicates your level of confidence in response to each statement about nurse-initiated care on the left. ‘0’ indicates no confidence. ‘10’ indicates complete confidence.

| *As of today, I am confident that I am able to appropriately and autonomously initiate the below care when clinically indicated*  (in relation to **nurse-initiated protocols** of care in your ED/MPS) | No Complete  Confidence Confidence | | | | | | | | | | |
| --- | --- | --- | --- | --- | --- | --- | --- | --- | --- | --- | --- |
|  | 0 | 1 | 2 | 3 | 4 | 5 | 6 | 7 | 8 | 9 | 10 |
| 1. Identify the need to start nurse-initiated care |  |  |  |  |  |  |  |  |  |  |  |
| 1. Initiate **pathology** investigations using nurse-initiated protocols |  |  |  |  |  |  |  |  |  |  |  |
| 1. Initiate **radiology** investigations using nurse-initiated protocols |  |  |  |  |  |  |  |  |  |  |  |
| 1. Initiate **opioid analgesia** using nurse-initiated protocols |  |  |  |  |  |  |  |  |  |  |  |
| 1. Initiate **simple analgesia** using nurse-initiated protocols eg paracetamol etc |  |  |  |  |  |  |  |  |  |  |  |
| 1. Initiate **bronchodilators** using nurse-initiated protocols |  |  |  |  |  |  |  |  |  |  |  |
| 1. Initiate **thromboembolytics** using nurse-initiated protocols |  |  |  |  |  |  |  |  |  |  |  |
| 1. Initiate interventions for **paediatric patients** |  |  |  |  |  |  |  |  |  |  |  |
| 1. Initiate **antiemetics** using nurse-initiated protocols |  |  |  |  |  |  |  |  |  |  |  |
| 1. Initiate **IV therapy** using nurse-initiated protocols |  |  |  |  |  |  |  |  |  |  |  |
| 1. Initiate **IV antibiotics** using nurse-initiated protocols |  |  |  |  |  |  |  |  |  |  |  |
| 1. Initiate **steroids** using nurse-initiated protocols |  |  |  |  |  |  |  |  |  |  |  |

## Instrument 4: Work environment based on the Practice Environment Scale of Nursing Workforce Index (PES-NWI)

| **Item No** | **Component items** | **Strongly disagree** |  | | **Strongly agree** |
| --- | --- | --- | --- | --- | --- |
|  | Adequate support services allow me to spend time with my patients | **1** | **2** | **3** | **4** |
|  | Doctors and nurses have good working relationships | 1 | 2 | 3 | 4 |
|  | ED managers that are supportive of the nurses | 1 | 2 | 3 | 4 |
|  | Active staff development or education programs for nurses. | 1 | 2 | 3 | 4 |
|  | Career development opportunity. | 1 | 2 | 3 | 4 |
|  | Opportunity for frontline nurses to participate in policy decisions. | 1 | 2 | 3 | 4 |
|  | Supervisors use mistakes as learning opportunities, not criticism | 1 | 2 | 3 | 4 |
|  | Enough time and opportunity to discuss patient care problems with other nurses. | 1 | 2 | 3 | 4 |
|  | Enough registered nurses to provide quality patient care | 1 | 2 | 3 | 4 |
|  | A nurse manager who is a good manager and leader. | 1 | 2 | 3 | 4 |
|  | A Director of Nursing who is highly visible and accessible to staff | 1 | 2 | 3 | 4 |
|  | Enough staff to get the work done | 1 | 2 | 3 | 4 |
|  | Praise and recognition for a job well done | 1 | 2 | 3 | 4 |
|  | High standards of nursing care are expected by the administration | 1 | 2 | 3 | 4 |
|  | A director of nursing equal in power and authority to other top level hospital executives | 1 | 2 | 3 | 4 |
|  | A lot of teamwork between nurses and doctors | 1 | 2 | 3 | 4 |
|  | Opportunities for advancement | 1 | 2 | 3 | 4 |
|  | A clear philosophy of nursing that pervades the patient care environment | 1 | 2 | 3 | 4 |
|  | Working with nurses who are clinically competent | 1 | 2 | 3 | 4 |
|  | A nurse manager who backs up the nursing staff in decision making, even if the conflict is with a doctor. | 1 | 2 | 3 | 4 |
|  | Administration that listens and responds to employee concerns | 1 | 2 | 3 | 4 |
|  | An active quality assurance program | 1 | 2 | 3 | 4 |
|  | Frontline nurses are involved in the internal governance of the hospital (e.g., practice and policy committees). | 1 | 2 | 3 | 4 |
|  | Collaboration (joint practice) between nurses and doctors. | 1 | 2 | 3 | 4 |
|  | A preceptor program for newly hired registered nurses | 1 | 2 | 3 | 4 |
|  | Nursing care is based on a nursing, rather than a medical, model. | 1 | 2 | 3 | 4 |
|  | Frontline nurses can participate on hospital and nursing committees | 1 | 2 | 3 | 4 |
|  | Nursing administrators consult with staff on daily problems and procedures | 1 | 2 | 3 | 4 |

**Adapted from Parker et al^1^**

## Instrument 5: Behavioural analysis

1. Do you think it is beneficial for all emergency nurses in NSW to use the same:
   1. standardised approach in the nurse-initiated treatment of patients? i.e. all ED have the same protocols available for initiation of care
   2. Standardised training
   3. Standardised accreditation

Scale 0-10

If No (0-5 on scale) additional questions –

Why is a standardised process not beneficial? (select all that apply)

1. Emergency departments have different resources so a standardised approach wont work
2. A single method will not suit all situations in the ED
3. Current practice is adequate
4. We all work differently, a single method doesn't suit all nurses
5. Other:
6. Do you think nurses should be responsible for commencing treatment on patients presenting to the ED?

Scale 0-10

Please explain:

**E**mergency nurse **P**rotocols **I**nitiating **C**are (**EPIC**) is a clinical framework to support the earlier **delivery** of standardised evidence-based treatment by emergency nurses for70 of the most common ED presentations. Earlier delivery of care ensures that fundamentals, such as pain relief, antibiotics, pathology and radiology, are initiated for patients prior to medical officer assessment.

1. Please indicate if you agree or disagree with the following statements about why you would or wouldn’t want to transition to EPICs.

|  |  | Strongly Disagree | | | | | | | Strongly  Agree | | | | |
| --- | --- | --- | --- | --- | --- | --- | --- | --- | --- | --- | --- | --- | --- |
|  |  |  | | | | | | | | | | | |
|  |  | 0 | 1 | 2 | 3 | 4 | 5 | 6 | | 7 | 8 | 9 | 10 |
|  | I am not interested in learning something new |  |  |  |  |  |  |  | |  |  |  |  |
|  | There are already too many protocols |  |  |  |  |  |  |  | |  |  |  |  |
|  | There is not enough time to change the way of working |  |  |  |  |  |  |  | |  |  |  |  |
|  | Patient’s will appreciate my being able to give them treatment earlier |  |  |  |  |  |  |  | |  |  |  |  |
|  | I don’t feel supported by the medical team in using nurse-initiated protocols |  |  |  |  |  |  |  | |  |  |  |  |
|  | The way we do things is fine, no need to change anything |  |  |  |  |  |  |  | |  |  |  |  |
|  | I don’t feel supported by management |  |  |  |  |  |  |  | |  |  |  |  |
|  | I will not have enough clinical support |  |  |  |  |  |  |  | |  |  |  |  |
|  | It is too hard to remember anything new |  |  |  |  |  |  |  | |  |  |  |  |
|  | It will not change the way I care for my patient |  |  |  |  |  |  |  | |  |  |  |  |
|  | The training will be time consuming |  |  |  |  |  |  |  | |  |  |  |  |

Please elaborate on why you will want to or not want to use EPIC (free text):

1. What do you think will help facilitate the implementation of EPIC in your department? (*select all that apply*)

|  | | Strongly Disagree | | | | | Strongly  Agree | | | | | | |
| --- | --- | --- | --- | --- | --- | --- | --- | --- | --- | --- | --- | --- | --- |
|  |  |  | | | | | | | | | | | |
|  |  | 0 | 1 | 2 | 3 | 4 | | 5 | 6 | 7 | 8 | 9 | 10 |
|  | Face to face education |  |  |  |  |  | |  |  |  |  |  |  |
|  | Support in the clinical environment to adjust |  |  |  |  |  | |  |  |  |  |  |  |
|  | Visual prompts to remind me (posters) |  |  |  |  |  | |  |  |  |  |  |  |
|  | Opportunity to ask questions |  |  |  |  |  | |  |  |  |  |  |  |
|  | personal feedback |  |  |  |  |  | |  |  |  |  |  |  |
|  | A policy |  |  |  |  |  | |  |  |  |  |  |  |
|  | Knowing that there are consequences if we don’t change |  |  |  |  |  | |  |  |  |  |  |  |
|  | Knowing that the change is being monitored |  |  |  |  |  | |  |  |  |  |  |  |
|  | Face to face education |  |  |  |  |  | |  |  |  |  |  |  |
|  | Support in the clinical environment to adjust |  |  |  |  |  | |  |  |  |  |  |  |
|  | Visual prompts to remind me (posters) |  |  |  |  |  | |  |  |  |  |  |  |
|  | A workable eMR |  |  |  |  |  | |  |  |  |  |  |  |
|  | Other |  |  |  |  |  | |  |  |  |  |  |  |

1. What do you think is the MOST important thing to help EPIC be implemented smoothly in your department? (free text)

***The EPIC Team wishes to thank you for your time and support***

# MEDICAL SURVEY

*The survey was developed and managed in RedCAP. The content of the online survey is below.*

| **Instrument** | **Items** | **Description** |
| --- | --- | --- |
| Participant characteristics | 6 | Participant demographics including role, years of general experience, and emergency medicine experience |
| Nurse-initiated care | 3 | perceptions of standardised nurse access to protocols (scale 0-100) and rationales |
| Satisfaction | 12 | satisfaction with nurse-initiated care (0-10 scale) |
| Behavioural diagnostics | 11 | transition to new nurse-initiated care protocols and potential impact of nurse-initiated care protocol implementation on medical staff |

## Instrument 1: Characteristics

Please select your current position

- ED Staff specialist
- Registrar
- Resident
- Intern
- CMO
- VMO
- Other_(please specify)___________

How many years have you worked as a medical officer?

How many years’ experience do you have working in an **emergency department**?

## Instrument 2: Nurse-initiated care

Nurse-initiated care can be defined as nursing staff autonomously providing care to patients prior to medical review. Examples are administering analgesia, bronchodilators, imaging and pathology requests.

Do you have nurse-initiated processes in your department?

- Yes
- No
- Unsure

Please indicate if you agree or disagree with the following statement:

All emergency nurses in NSW should use the same standardised approach for nurse-initiated care (Scale 0-100)

Why?

Why not?

If No 1 additional questions –

Why is a standardised process not required? (select all that apply)

1. Emergency departments have different resources
2. There is no benefit to a standardised process
3. A single method will not suit all situations in the ED
4. Current practice is adequate
5. Other - Please elaborate: (free text)

## Instrument 3: Satisfaction with nurse-initiated protocols

## Select the number which indicates your level of satisfaction with each of the items.

‘0’ indicates no satisfaction. ‘10’ indicates complete satisfaction. Put N/A if not applicable to your experience

| In the last week, how satisfied have you been with the following in relation to nurse-initiated protocols in the emergency department:  **Appropriateness of** | No  satisfaction | | | | | Complete  Satisfaction | | | | | | | N/A |
| --- | --- | --- | --- | --- | --- | --- | --- | --- | --- | --- | --- | --- | --- |
|  |  | | | | | | | | | | | |  |
| 1. The nurse-initiated protocols | 0 | 1 | 2 | 3 | 4 | | 5 | 6 | 7 | 8 | 9 | 10 | N/A |
| 1. Initiating **pathology** investigations | 0 | 1 | 2 | 3 | 4 | | 5 | 6 | 7 | 8 | 9 | 10 | N/A |
| 1. Initiating **radiology** investigations | 0 | 1 | 2 | 3 | 4 | | 5 | 6 | 7 | 8 | 9 | 10 | N/A |
| 1. Initiating **opioid analgesia** | 0 | 1 | 2 | 3 | 4 | | 5 | 6 | 7 | 8 | 9 | 10 | N/A |
| 1. Initiating **simple analgesia eg non-opioid medication** | 0 | 1 | 2 | 3 | 4 | | 5 | 6 | 7 | 8 | 9 | 10 | N/A |
| 1. Initiating **broncholdialators** | 0 | 1 | 2 | 3 | 4 | | 5 | 6 | 7 | 8 | 9 | 10 | N/A |
| 1. Initiating **thromboembolytics** | 0 | 1 | 2 | 3 | 4 | | 5 | 6 | 7 | 8 | 9 | 10 | N/A |
| 1. Initiate interventions for **paediatric patients** | 0 | 1 | 2 | 3 | 4 | | 5 | 6 | 7 | 8 | 9 | 10 | N/A |
| 1. Initiate **antiemetics** | 0 | 1 | 2 | 3 | 4 | | 5 | 6 | 7 | 8 | 9 | 10 | N/A |
| 1. Initiate **IV therapy** | 0 | 1 | 2 | 3 | 4 | | 5 | 6 | 7 | 8 | 9 | 10 | N/A |
| 1. Initiate **IV antibiotics** | 0 | 1 | 2 | 3 | 4 | | 5 | 6 | 7 | 8 | 9 | 10 | N/A |
| 1. Initiate **steroids** | 0 | 1 | 2 | 3 | 4 | | 5 | 6 | 7 | 8 | 9 | 10 | N/A |

**Instrument 4: Transition to EPIC**

**E**mergency nurse **P**rotocols **I**nitiating **C**are (**EPIC**) is a clinical framework to support the earlier **delivery** of standardised evidence-based treatment by emergency nurses for 74 of the most common ED presentations. Earlier delivery of care ensures that fundamentals, such as pain relief, antibiotics, pathology and radiology, are initiated for patients prior to medical officer assessment.

1. Please indicate if you agree or disagree with the following statements about why you **would or wouldn’t** want to transition to EPICs.

|  | Strongly Disagree | | | | | | Strongly  Agree | | | | | |
| --- | --- | --- | --- | --- | --- | --- | --- | --- | --- | --- | --- | --- |
|  |  | | | | | | | | | | | |
|  | 0 | 1 | 2 | 3 | 4 | 5 | | 6 | 7 | 8 | 9 | 10 |
| It will be more work for me later |  |  |  |  |  |  | |  |  |  |  |  |
| I don’t feel management will support the transition |  |  |  |  |  |  | |  |  |  |  |  |
| It will be better for patient care |  |  |  |  |  |  | |  |  |  |  |  |
| It would be easier for me to just do it myself |  |  |  |  |  |  | |  |  |  |  |  |
| It will save me time for patient care |  |  |  |  |  |  | |  |  |  |  |  |
| It will reduce the patient time to diagnosis and treatment |  |  |  |  |  |  | |  |  |  |  |  |
| The way we do things is fine, no need to change anything |  |  |  |  |  |  | |  |  |  |  |  |
| It will increase my administrative workload |  |  |  |  |  |  | |  |  |  |  |  |

***The EPIC Team wishes to thank you for your time and support***

1. Parker D, Tuckett A, Eley R, Hegney D. Construct validity and reliability of the Practice Environment Scale of the Nursing Work Index for Queensland nurses. *Int J Nurs Pract*. Aug 2010;16(4):352-8. doi:10.1111/j.1440-172X.2010.01851.x
