# Supplementary material for: Identifying Barriers and Enablers for Nurse‐Initiated Care for Designing Implementation at Scale in Australian Emergency Departments: A Mixed Methods Study
Source: J Clin Nurs. 2025 Feb 19;34(7):2718–36. doi: 10.1111/jocn.17693 (PMC12181148; doi:10.1111/jocn.17693)
Supplement: Supplementary file 2 — Appendix S2. [file JOCN-34-2718-s003.docx]

# Supplementary data analysis

# Table 1. Quantitative responses to the nursing survey and preliminary barriers (B) and enablers (E) based on agreement score.

|  | **ALL** | **Cluster1** | **Cluster2** | **Cluster3** | **Cluster4** | **p-value^** |
| --- | --- | --- | --- | --- | --- | --- |
|  | 602 | 109 | 187 | 125 | 181 |  |
| **Should nurses be responsible for INITIATING treatment of patients presenting to the ED when appropriate (scale 0-100)?** (E) **Median (IQR)** | 80.0 (66.0, 98.0) | 96.5 (78.0, 100.0) | 83.0 (67.0, 99.0) | 77.0 (68.0, 92.0) | 75.0 (51.0, 93.0) | **<0.001** |
| **Do you have nurse-initiated protocols in your ED?** (E)  **Yes - n (%)** | 570 (94.7%) | 103 (94.5%) | 180 (96.3%) | 119 (95.2%) | 168 (92.8%) | 0.523 |
| **What nurse-initiated care are you accredited to provide in your ED?**  **Yes - n (%)** | 570 | 103 | 180 | 119 | 168 |  |
| Nurse-initiated x-ray (B) | 159 (27.9%) | 39 (37.9%) | 37 (20.6%) | 49 (41.2%) | 34 (20.2%) | **<0.001** |
| Nurse-initiated medications (NIM) (E) | 489 (85.8%) | 68 (66.0%) | 170 (94.4%) | 106 (89.1%) | 145 (86.3%) | **<0.001** |
| Noneⴕ (E) | 34 (6.0%) | 7 (6.8%) | 0(0.0%) | 9 (7.6%) | 18 (10.7%) | **<0.001** |
| **What do you like about current nurse-initiated protocols? (select any that apply)**  **N(%)** | | | | | | |
| Improves patient care (E) | 504 (83.7%) | 88 (80.7%) | 168 (89.8%) | 97 (77.6%) | 151 (83.4%) | **0.026** |
| Speeds things up for my patient (E) | 498 (82.7%) | 91 (83.5%) | 156 (83.4%) | 105 (84.0%) | 146 (80.7%) | 0.852 |
| Make a difference in patient care (E) | 429 (71.3%) | 84 (77.1%) | 140 (74.9%) | 81 (64.8%) | 124 (68.5%) | 0.102 |
| Autonomy | 388 (64.5%) | 76 (69.7%) | 118 (63.1%) | 83 (66.4%) | 111 (61.3%) | 0.484 |
| Patient appreciates it (E – Cluster2 only) | 378 (62.8%) | 67 (61.5%) | 132 (70.6%) | 73 (58.4%) | 106 (58.6%) | 0.061 |
| Professional development/ satisfaction | 326 (54.2%) | 68 (62.4%) | 90 (48.1%) | 65 (52.0%) | 103 (56.9%) | 0.090 |
| Acknowledges or validates my practice | 310 (51.5%) | 61 (56.0%) | 94 (50.3%) | 62 (49.6%) | 93 (51.4%) | 0.761 |
| Protocols are easy to find / access | 302 (50.2%) | 51 (46.8%) | 96 (51.3%) | 58 (46.4%) | 97 (53.6%) | 0.540 |
| Our protocols provide clear indications for use | 281 (46.7%) | 46 (42.2%) | 86 (46.0%) | 59 (47.2%) | 90 (49.7%) | 0.659 |
| Medical staff appreciate it | 266 (44.2%) | 55 (50.5%) | 82 (43.9%) | 54 (43.2%) | 75 (41.4%) | 0.503 |
| Our protocols are easy to follow | 229 (38.0%) | 41 (37.6%) | 74 (39.6%) | 47 (37.6%) | 67 (37.0%) | 0.963 |
| **What do you not like about current nurse-initiated protocols? (select any that apply)**  **N (%)** | | | | | | |
| Interventions are too limited (E) | 216 (35.9%) | 61 (56.0%) | 54 (28.9%) | 35 (28.0%) | 66 (36.5%) | **<0.001** |
| Difficult to access (B) | 99 (16.4%) | 15 (13.8%) | 35 (18.7%) | 27 (21.6%) | 22 (12.2%) | 0.106 |
| Too vague | 78 (13.0%) | 23 (21.1%) | 21 (11.2%) | 11 (8.8%) | 23 (12.7%) | 0.032 |
| Too many steps involved | 71 (11.8%) | 8 (7.3%) | 30 (16.0%) | 13 (10.4%) | 20 (11.0%) | 0.130 |
| I cannot record in eMR which protocol I used | 61 (10.1%) | 12 (11.0%) | 13 (7.0%) | 20 (16.0%) | 16 (8.8%) | 0.065 |
| I do not have time to use them ⴕ | 61 (10.1%) | 3 (2.8%) | 21 (11.2%) | 16 (12.8%) | 21 (11.6%) | **0.020** |
| I do not know when to use them | 44 (7.3%) | 6 (5.5%) | 14 (7.5%) | 14 (11.2%) | 10 (5.5%) | 0.243 |
| Medical staff do not support ⴕ | 32 (5.3%) | 12 (11.0%) | 7 (3.7%) | 2 (1.6%) | 11 (6.1%) | **0.011** |
| Too complex | 31 (5.1%) | 5 (4.6%) | 6 (3.2%) | 11 (8.8%) | 9 (5.0%) | 0.177 |
| Does not help patient ⴕ | 6 (1.0%) | 1 (0.9%) | 1 (0.5%) | 1 (0.8%) | 3 (1.7%) | 0.818 |
| Other | 58 (9.6%) | 14 (12.8%) | 20 (10.7%) | 14 (11.2%) | 10 (5.5%) | 0.145 |
| **As of today, I am confident that I am able to appropriately and autonomously initiate the below care when clinically indicated (in relation to nurse-initiated protocols**  **Median (IQR: Q1 to Q3)** | | | | | | |
| Initiate simple analgesia using nurse-initiated protocols eg paracetamol etc (E) | 10.0 (8.0 to 10.0) | 10.0 (9.0 to 10.0) | 10.0 (9.0 to 10.0) | 10.0 (8.0 to 10.0) | 10.0 (8.0 to 10.0) | **0.030** |
| Identify the need to start nurse-initiated care (E) | 8.0 (7.0 to 10.0) | 9.0 (8.0 to 10.0) | 9.0 (7.0 to 10.0) | 8.0 (7.0 to 10.0) | 8.0 (6.0 to 10.0) | **<0.001** |
| Initiate pathology investigations using nurse-initiated protocols (E) | 8.0 (6.0 to 10.0) | 8.0 (6.0 to 10.0) | 8.0 (6.0 to 10.0) | 8.0 (7.0 to 10.0) | 8.0 (6.0 to 10.0) | 0.848 |
| Initiate bronchodilators using nurse-initiated protocols (E) | 8.0 (6.0 to 10.0) | 9.0 (7.0 to 10.0) | 9.0 (7.0 to 10.0) | 7.0 (4.0 to 9.0) | 7.0 (5.0 to 10.0) | **<0.001** |
| Initiate antiemetics using nurse-initiated protocols (E) | 8.0 (6.0 to 10.0) | 9.0 (8.0 to 10.0) | 9.0 (8.0 to 10.0) | 8.0 (5.0 to 9.0) | 7.0 (5.0 to 9.0) | **<0.001** |
| Initiate opioid analgesia using nurse-initiated protocols (B - Cluster3, E other sites) | 8.0 (5.0 to 10.0) | 8.0 (5.0 to 10.0) | 8.0 (6.0 to 10.0) | 6.0 (3.0 to 9.0) | 7.0 (4.5 to 9.5) | **<0.001** |
| Initiate IV therapy using nurse-initiated protocols (E) | 8.0 (4.0 to 10.0) | 9.0 (6.0 to 10.0) | 7.0 (5.0 to 10.0) | 8.0 (3.0 to 9.0) | 7.0 (4.0 to 9.0) | **0.004** |
| Initiate radiology investigations using nurse-initiated protocols (B) | 6.0 (3.0 to 9.0) | 7.0 (4.0 to 9.0) | 6.0 (3.0 to 8.0) | 7.0 (3.0 to 9.0) | 5.0 (2.0 to 8.0) | **<0.001** |
| Initiate interventions for paediatric patients (B) | 6.0 (3.0 to 9.0) | 7.0 (5.0 to 9.0) | 7.0 (5.0 to 10.0) | 5.0 (2.0 to 8.0) | 4.0 (0.0 to 8.0) | **<0.001** |
| Initiate IV antibiotics using nurse-initiated protocols (B) | 5.0 (1.0 to 8.0) | 7.0 (3.0 to 10.0) | 5.0 (2.0 to 9.0) | 1.0 (0.0 to 5.0) | 4.0 (1.0 to 7.0) | **<0.001** |
| Initiate steroids using nurse-initiated protocols (B) | 5.0 (1.0 to 8.0) | 7.0 (4.0 to 9.0) | 6.0 (2.0 to 9.0) | 2.0 (0.0 to 6.0) | 4.5 (1.0 to 7.0) | **<0.001** |
| Initiate thromboembolics using nurse-initiated protocols (B) | 4.0 (0.0 to 7.0) | 5.0 (2.0 to 8.0) | 5.0 (1.0 to 8.0) | 1.0 (0.0 to 5.0) | 3.0 (0.0 to 6.0) | **<0.001** |
| **Please indicate if you agree or disagree with the following statements about why you would or wouldn’t want the same protocols, training and accreditation across NSW 0 = Strongly Disagree to 10 = Strongly Agree** | | | | | | |
| **Barriers Agree - n (%)** |  |  |  |  |  |  |
| A single method will suit all situations in the ED | 205 (39.0%) | 46 (48.9%) | 54 (31.2%) | 31 (32.0%) | 74 (46.0%) | **0.029** |
| The training can be standardised | 206 (39.2%) | 41 (43.6%) | 54 (31.2%) | 36 (37.1%) | 75 (46.6%) | 0.126 |
| A standard approach will work | 279 (53.1%) | 57 (60.6%) | 82 (47.4%) | 54 (55.7%) | 86 (53.4%) | 0.382 |
| I will have enough clinical support | 330 (62.9%) | 68 (72.3%) | 110 (63.6%) | 55 (56.7%) | 97 (60.2%) | 0.081 |
| There needs to be more protocols | 341 (65.0%) | 82 (87.2%) | 106 (61.3%) | 53 (54.6%) | 100 (62.1%) | **<0.001** |
| The training will not be time-consuming | 348 (66.3%) | 69 (73.4%) | 107 (61.8%) | 67 (69.1%) | 105 (65.2%) | 0.433 |
| I feel supported by the medical team in using nurse-initiated protocols | 349 (66.5%) | 59 (62.8%) | 122 (70.5%) | 72 (74.2%) | 96 (59.6%) | **0.026** |
| I feel supported by management | 353 (67.2%) | 70 (74.5%) | 121 (69.9%) | 58 (59.8%) | 104 (64.6%) | 0.397 |
| **Agree or disagree about statements regarding implementation of ECATs at your site** | | | | | | |
| **Enablers nursing Agree - n (%)** |  |  |  |  |  |  |
| Patients will appreciate my being able to give them treatment earlier | 468 (89.1%) | 83 (88.3%) | 159 (91.9%) | 88 (90.7%) | 138 (85.7%) | 0.581 |
| It is easy to remember something new | 449 (85.5%) | 86 (91.5%) | 149 (86.1%) | 85 (87.6%) | 129 (80.1%) | 0.190 |
| I am interested in learning something new | 444 (84.6%) | 85 (90.4%) | 143 (82.7%) | 87 (89.7%) | 129 (80.1%) | 0.098 |
| It will change the way I care for my patient | 434 (82.7%) | 82 (87.2%) | 138 (79.8%) | 82 (84.5%) | 132 (82.0%) | 0.515 |
| The way we do things needs to change | 431 (82.1%) | 85 (90.4%) | 133 (76.9%) | 83 (85.6%) | 130 (80.7%) | 0.142 |
| Confidence to Identify the need to start nurse-initiated care | 8.0 (7.0,10.0) | 9.0 (8.0, 10.0) | 9.0 (7.0, 10.0) | 8.0 (7.0, 0.0) | 8.0 (6.0, 10.0) | **<0.001** |
| Confidence to Initiate pathology investigations using nurse-initiated protocols | 8.0 (6.0 10.0) | 8.0 (6.0, 10.0) | 8.0 (6.0, 10.0) | 8.0 (7.0, 0.0) | 8.0 (6.0, 10.0) | 0.848 |
| Current practice is inadequate | 362 (69.0%) | 75 (79.8%) | 114 (65.9%) | 67 (69.1%) | 106 (65.8%) | 0.094 |
| There is enough time to change the way of working | 345 (65.7%) | 77 (81.9%) | 110 (63.6%) | 60 (61.9%) | 98 (60.9%) | **0.013** |
|  |  |  |  |  |  |  |

^ Chi-squared test or ⴕiterms: Fisher's Exact Test for categorical variables, Kruskal–Wallis test for continuous variables. Significant result in bold

# Table 2. Nursing Practice Environment Scale of the Nursing Work Index calculated as Subscales [1]

| **Nursing Work Index: Subscales score = 1-4**  Mean (Sd) | **All** | **Cluster1** | **Cluster2** | **Cluster3** | **Cluster4** | **P-value^** |
| --- | --- | --- | --- | --- | --- | --- |
| Nurse participation in hospital affairs (B) | 2.65 (0.59) | 2.73 (0.57) | 2.62 (0.62) | 2.48 (0.55) | 2.74 (0.57) | **0.002** |
| Nursing foundations for quality care (B) | 2.98 (0.45) | 2.97 (0.48) | 3.00 (0.43) | 2.84 (0.40) | 3.05 (0.47) | **<0.001** |
| Nurse manager ability and leadership and support of nurses (B) | 2.99 (0.59) | 3.22 (0.51) | 2.98 (0.61) | 2.75 (0.56) | 3.01 (0.59) | **<0.001** |
| Staffing and resource adequacy (B) | 2.39 (0.68) | 2.58 (0.61) | 2.45 (0.64) | 2.16 (0.63) | 2.37 (0.74) | **<0.001** |
| Collegial nurse-physician relationships (E) | 3.23 (0.51) | 3.27 (0.48) | 3.39 (0.46) | 3.10 (0.53) | 3.13 (0.53) | **<0.001** |

1. Swiger PA, Patrician PA, Miltner RSS, Raju D, Breckenridge-Sproat S, Loan LA. The Practice Environment Scale of the Nursing Work Index: An updated review and recommendations for use. Int J Nurs Stud. 2017;74:76-84.

^F-test of one-way ANOVA, Barriers (B) when score <3 and Enabler (E) score > 3

# Table 3. Quantitative responses to the medical survey and preliminary barriers (B) and enablers (E) based on score agreement with statements

|  | **All** | **Cluster1** | **Cluster2** | **Cluster3** | **Cluster4** | p-value |
| --- | --- | --- | --- | --- | --- | --- |
|  | 245 | 31 | 60 | 79 | 75 |  |
| **Should all emergency nurses have access to and training in the same protocols? Scale 0-100 (E)** | 86.0 (72.0 to 100.0) | 89.0 (72.0 to 100.0) | 84.5 (70.0 to 100.0) | 84.0 (73.0 to 100.0) | 90.0 (72.0 to 100.0) | 0.832 |
| **If <50 – select reason:** |  |  |  |  |  |  |
| Emergency departments have different resources | 17 (6.9%) | 1 (3.2%) | 7 (11.7%) | 5 (6.3%) | 4 (5.3%) |  |
| There is no benefit to a standardised process | 1 (0.4%) |  | 1 (1.7%) |  |  |  |
| A single method will not suit all situations in the ED | 24 (9.8%) |  | 8 (13.3%) | 8 (10.1%) | 8 (10.7%) |  |
| Current practice is adequate | 3 (1.2%) |  |  | 1 (1.3%) | 2 (2.7%) |  |
| **How satisfied have you been with the following in relation to nursing practice in the ED**  **(Scale 0-10)** | | | | | | |
| Simple analgesia e.g. non-opioid medication (E) | 9.0 (8.0 to 10.0) | 9.0 (8.0 to 10.0) | 8.5 (7.0 to 10.0) | 8.0 (7.0 to 10.0) | 9.0 (8.0 to 10.0) | 0.604 |
| Protocols in general (E) | 8.0 (7.0 to 9.0) | 7.0 (6.0 to 9.0) | 8.0 (7.0 to 8.5) | 8.0 (6.0 to 9.0) | 8.0 (7.0 to 9.0) | 0.691 |
| Bronchodilators (E) | 8.0 (6.0 to 9.0) | 8.0 (6.0 to 10.0) | 8.0 (7.0 to 9.5) | 7.0 (5.0 to 9.0) | 8.0 (7.0 to 9.0) | 0.088 |
| Antiemetics (E) | 8.0 (7.0 to 10.0) | 8.0 (7.0 to 10.0) | 8.0 (7.0 to 10.0) | 8.0 (6.0 to 9.0) | 8.0 (7.0 to 10.0) | 0.096 |
| Pathology investigations | 7.0 (6.0 to 8.0) | 7.0 (6.0 to 9.0) | 6.5 (5.0 to 8.0) | 7.0 (7.0 to 8.0) | 8.0 (6.0 to 8.0) | 0.086 |
| Radiology investigations | 7.0 (5.0 to 8.0) | 7.0 (6.0 to 8.0) | 7.0 (4.0 to 8.0) | 8.0 (7.0 to 8.0) | 7.0 (5.0 to 8.0) | 0.083 |
| Opioid analgesia | 7.0 (5.0 to 8.0) | 7.0 (6.0 to 9.0) | 7.0 (4.0 to 9.0) | 7.0 (4.0 to 8.0) | 7.0 (6.0 to 9.0) | 0.134 |
| IV therapy | 7.0 (5.0 to 8.0) | 7.0 (5.0 to 9.0) | 6.5 (3.5 to 8.0) | 6.0 (5.0 to 8.0) | 7.0 (6.0 to 9.0) | **0.020** |
| Interventions for paediatric patients (B) | 6.0 (5.0 to 8.0) | 6.0 (5.0 to 8.0) | 7.0 (5.0 to 8.0) | 6.0 (5.0 to 8.0) | 5.0 (3.0 to 7.0) | **0.030** |
| Thromboembolics (B) | 5.0 (0.0 to 6.0) | 5.0 (2.0 to 6.0) | 4.5 (0.0 to 6.0) | 4.0 (0.0 to 5.0) | 5.0 (0.0 to 5.0) | 0.304 |
| IV antibiotics (B) | 5.0 (2.0 to 7.0) | 5.0 (3.0 to 8.0) | 5.0 (1.0 to 7.5) | 5.0 (2.0 to 6.0) | 5.0 (2.0 to 7.0) | 0.392 |
| Steroids (B) | 5.0 (2.0 to 7.0) | 6.0 (2.0 to 7.0) | 5.0 (1.0 to 7.0) | 5.0 (2.0 to 6.0) | 5.0 (1.0 to 7.0) | 0.337 |
|  |  |  |  |  |  |  |
| **With regards to nurses initiating care…. score the following statements on the scale from 0 =Strongly Disagree –> 10 = Strongly Agree** | | | | | |  |
| **Enablers n(%)** |  |  |  |  |  |  |
| I fully support nurses initiating care ⴕ | 226 (92.2%) | 28 (90.3%) | 54 (90.0%) | 73 (92.4%) | 71 (94.7%) | 0.687 |
| It will be better for patient care ⴕ | 219 (89.4%) | 27 (87.1%) | 54 (90.0%) | 71 (89.9%) | 67 (89.3%) | 0.510 |
| It will save me time ⴕ | 217 (88.6%) | 28 (90.3%) | 49 (81.7%) | 70 (88.6%) | 70 (93.3%) | 0.439 |
| It will reduce the patient time to treatment ⴕ | 210 (85.7%) | 27 (87.1%) | 49 (81.7%) | 67 (84.8%) | 67 (89.3%) | 0.699 |
| It will reduce the patient time to diagnosis ⴕ | 201 (82.0%) | 25 (80.6%) | 49 (81.7%) | 61 (77.2%) | 66 (88.0%) | 0.557 |
| Management will support the transition | 188 (76.7%) | 20 (64.5%) | 42 (70.0%) | 64 (81.0%) | 62 (82.7%) | 0.367 |
| All emergency nurses should have access to & training in the same protocols | 86.0 (72.0, 100.0) | 89.0 (72.0, 100.0) | 84.5 (70.0, 100.0) | 84.0 (73.0, 100.0) | 90.0 (72.0, 100.0) | 0.832 |
| **Barriers n(%)** |  |  |  |  |  |  |
| It will increase my administrative workload | 49 (20.0%) | 9 (29.0%) | 8 (13.3%) | 17 (21.5%) | 15 (20.0%) | 0.549 |
| It would be easier for me to just do it myself | 48 (19.6%) | 12 (38.7%) | 7 (11.7%) | 14 (17.7%) | 15 (20.0%) | 0.091 |
| It is beyond the scope of nursing practice | 41 (16.7%) | 8 (25.8%) | 10 (16.7%) | 13 (16.5%) | 10 (13.3%) | 0.213 |
| It will be more work for me later | 33 (13.5%) | 10 (32.3%) | 3 (5.0%) | 10 (12.7%) | 10 (13.3%) | **0.007** |
|  |  |  |  |  |  |  |

^ Chi-squared test or ⴕ iterms: Fisher's Exact Test for categorical variables, Kruskal–Wallis test for continuous variables.

# Table 4. Categories and sub-categories from free text survey responses regarding HIRAID® implementation with indication if preliminary barrier (B) or enabler (E). Number of references from individuals are listed and some sample quotes

| Name | Sites | Ref |
| --- | --- | --- |
| *Category 1. Staff are supportive of new nurse-initiated care protocols citing considerable benefits to patients and staff (E)* | ***8*** | ***1107*** |
| There are multiple benefits to nurse-initiated care *(E)* | 8 | 849 |
| -Advancement of nursing as a profession improving nursing care | 7 | 219 |
| -Enables medical staff in patient care | 6 | 39 |
| -Improved departmental performance and work environment | 8 | 237 |
| -Improved patient care and experience | 8 | 354 |
| There is wide support for the use of nurse-initiated care *(E)* | 8 | 258 |
| -Nurses are capable and well-placed to initiate care | 6 | 37 |
| -Positive prior experiences with nurse-initiated care | 7 | 68 |
| -Supportive of nurse-initiated care change | 8 | 153 |
| *Category 2. The pre-existing nurse-initiated care protocols are restrictive and lack standardisation, so change, that considers nursing work practices is needed (n=505). (E)* | ***8*** | ***505*** |
| Design of protocols will impact use *(E)* | 8 | 325 |
| -Protocol designed well with thorough content | 7 | 34 |
| -Protocols that lack clarity hinder use | 7 | 93 |
| -Protocols that are too limited hinder use | 8 | 198 |
| Easy access and use of protocols will impact use *(E)* | 5 | 180 |
| -Process that is complex hinders use | 5 | 88 |
| -Protocols difficult to access hinder use | 4 | 92 |
| *Category 3. Successful and safe use of an expanded and standardised series of nurse-initiated care will need robust, face-to-face clinician-focused education and training in protected non-clinical time (n=641).* | ***8*** | ***641*** |
| Comprehensive clinician-focused education and training are crucial to implementation *(E)* | 8 | 335 |
| -Adequate and protected time for education and training needed | 4 | 136 |
| -Comprehensive education and or training needed to support | 8 | 199 |
| In-person education is required *(E)* | 8 | 306 |
| -Delivery of education needs to be in person and consider staff learning needs | 8 | 306 |
| *Category 4. Successful implementation of nurse-initiated care needs a robust, clinician-focused multi-methods strategy that enables nurses and includes support from management, medical and allied health staff (n=770).* | ***8*** | ***770*** |
| Multi-method implementation supports are needed (E) | 8 | 608 |
| -Clinical support needed for change | 6 | 143 |
| -Need thorough two-way communication with staff on change | 5 | 116 |
| -Social supports needed from management and senior staff | 7 | 170 |
| -well-planned change | 7 | 178 |
| A lack of human and physical resources hinders implementation (B) | 8 | 162 |
| -High workload and time constraints in the ED hinder use | 7 | 51 |
| -Limited resources hinder use | 7 | 28 |
| -Staff culture needs to embrace change | 5 | 15 |
| -Staffing and skill mix inadequate to support change | 8 | 68 |
| *Category 5. There may be risks associated with nurse-initiated protocol use so risk mitigation strategies should be instigated.* | ***8*** | ***438*** |
| Concerns for potential risk to patient safety (B) | 8 | 280 |
| -Change should consider the context of the sites | 6 | 46 |
| -Concern Inappropriate care may be given | 8 | 179 |
| -Unwanted change to the nursing role | 5 | 39 |
| Concerns for potential risk to staff safety (B) | 8 | 158 |
| -Increased responsibility for nurses | 7 | 76 |
| -The experience of nurse will impact the success of nurse-initiated care | 8 | 82 |
